# stMixer for Scalable Mosaic Integration and Label Transfer in Spatial Histology and Multi‐Omics

**DOI:** 10.1002/advs.75905

**Published:** 2026-06-02

**Authors:** Qixing Yang, Yan Wang, Luonan Chen, Chunman Zuo

**Affiliations:** ^1^ College of Computer Science and Technology Key Laboratory of Symbolic Computation and Knowledge Engineering of Ministry of Education Jilin University Changchun China; ^2^ State Key Laboratory of Biocontrol Innovation Center for Evolutionary Synthetic Biology School of Life Sciences Sun Yat‐sen University Guangzhou China; ^3^ School of Mathematical Sciences and School of AI Shanghai Jiao Tong University Shanghai China; ^4^ Key Laboratory of Systems Health Science of Zhejiang Province School of Life Science Hangzhou Institute for Advanced Study University of Chinese Academy of Sciences Chinese Academy of Sciences Hangzhou China; ^5^ Tianfu Jincheng Laboratory Chengdu China

**Keywords:** Self‐looped cross‐attention, Spatial histology and multi‐omics integration, Label transfer, Mosaic integration

## Abstract

Integrating spatial histology with multi‐slide, multi‐omics data is essential for deciphering tissue architecture and cellular dynamics at high resolution. However, incomplete modality overlap across sections hinders coherent integration and cross‐condition analysis. Here, we present stMixer, an unsupervised framework that (i) employs self‐looped cross‐attention to jointly encode histological, molecular, and spatial features; (ii) implements a multi‐modal metric learning module to achieve biologically coherent integration across sections; and (iii) uses a graph‐guided, cluster‐level voting algorithm to enable anatomically faithful label propagation. Benchmarking across six spatial modalities demonstrates that stMixer achieves superior scalability and accuracy in dimensionality reduction, batch correction, and label transfer. The framework accommodates large, heterogeneous datasets across tissues, species, and technologies. We further showcase its versatility in mosaic integration, pseudo‐time inference, and cross‐tissue knowledge transfer. Notably, stMixer uncovers transient thymic states overlooked by competing methods, resolves fine‐grained cortical microstructures, and corrects anatomical mis‐annotations through integration with single‐cell reference. stMixer is available at https://github.com/YQX‐code/stMixer/.

## Graphical abstract

stMixer is an unsupervised framework for scalable integration and label transfer across spatial histology and multi‐slide multi‐omics data with incomplete modality overlap. It combines self‐looped cross‐attention, multimodal metric learning, and graph‐guided cluster voting to align heterogeneous sections, correct batch effects, and propagate anatomically coherent labels, uncovering tissue domains, transitional states, and fine‐grained microstructures.

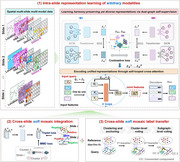

## 1 Introduction

The widespread application of single‐cell and spatial omics technologies has led to the accumulation of vast datasets across diverse tissues, capturing gene expression, chromatin accessibility, and protein profiles in spatially resolved contexts [1, 2, 3, 4]. In parallel, emerging spatial multi‐omics platforms—such as MISAR‐seq [5], DBiT‐seq [6], SM‐Omics [7], and SPOTS [8]—now enable the simultaneous profiling of multiple molecular layers within intact tissue architectures [9], generating multi‐modal data across millions of cells. These rich data resources offer unprecedented opportunities to reconstruct tissue architecture, delineate developmental trajectories, and decode the spatial basis of disease heterogeneity [10]. However, unlocking their full potential is impeded by key computational challenges, including the scalable integration of diverse modalities, alignment of partially overlapping sections, and robust label propagation from reference atlases.

Despite recent progress, three major limitations remain. (1) Integrating multiple omics layers within a single section is increasingly challenging, as modality heterogeneity and data scale amplify noise, sparsity, and alignment complexity. Existing tools, e.g., Seurat [11], MultiVI [12], totalVI [13], STAGATE [14], CellCharter [15], SpatialGlue [16], SSGATE [17], MISO [18], 3d‐OT [19], and COSMOS [20], are often restricted to fixed modality combinations or require labor‐intensive manual curation. (2) Cross‐slide mosaic integration is essential for reconstructing tissue‐wide landscapes but is limited by inconsistent modality availability. Single‐cell tools like MaxFuse [21] support heterogeneous alignment but neglect spatial context. To date, SpaMosaic [22] is the only method handling variable omics compositions across sections, though its graph‐based design incurs high memory costs and limits scalability. (3) Accurate label transfer from single‐cell or spatial references to new spatial multi‐omics datasets remains unsolved, due to batch effects and anatomical misalignments. Together, these challenges call for a unified, scalable framework that flexibly integrates heterogeneous modalities within and across slides, adapts to incomplete data compositions, and ensures anatomically coherent label propagation.

To address these unmet needs, we present stMixer, a unified model designed to integrate and annotate spatial multi‐omics data across slides and modalities. stMixer (i) unifies spatial, histological, and molecular signals via a self‐looped cross‐attention mechanism guided by a learnable Gaussian prior; (ii) performs spatial‐, profile‐, or histological‐informed metric learning, jointly optimized through triplet and maximum mean discrepancy (MMD) losses, to align biologically similar cells while preserving structural distinctions; and (iii) propagates annotations using a two‐stage, graph‐guided cluster‐level voting strategy that ensures anatomical coherence across sections.

Applied to both synthetic benchmarks and nine real‐world spatial datasets generated by five technologies across lymph node, thymus, brain, spleen, and breast cancer, stMixer consistently outperforms existing methods in spatial domain delineation, cross‐sectional integration, and label transfer. It reveals unrecognized thymic cell states, resolves fine‐grained cortical microstructures, and corrects annotation errors in anatomically ambiguous regions, underscoring its power to decode the intricate, multimodal architecture of biological tissues. Together, these results establish stMixer as a scalable and versatile framework for decoding complex tissue organization from increasingly diverse spatial omics data, paving the way for next‐generation reference atlases and cross‐condition biological discovery.

## 2 Result

### 2.1 Overview of stMixer

**FIGURE 1:**
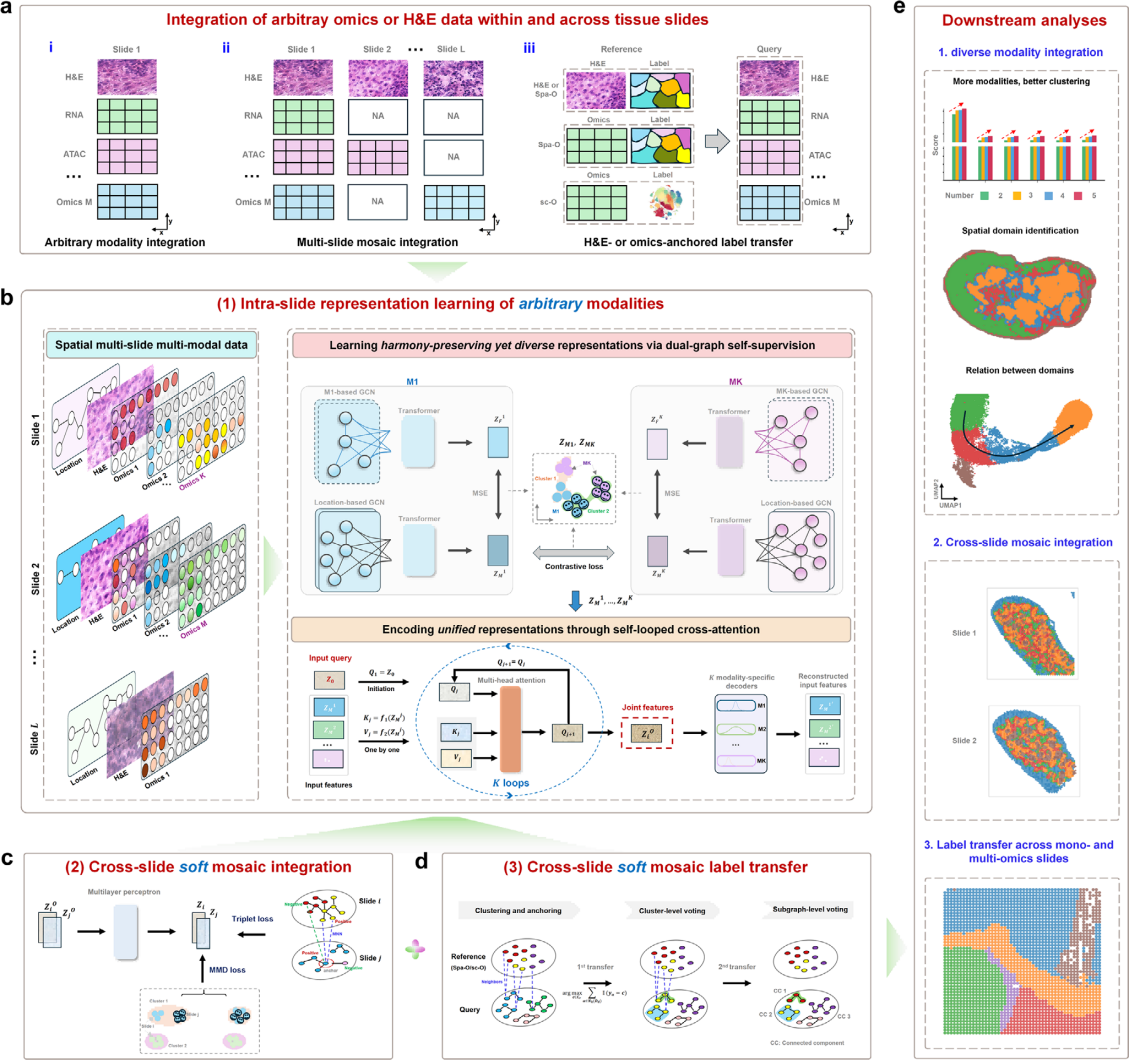
Overview of stMixer. (a) stMixer supports the integration of diverse modalities (including H&E and omics), multi‐slide mosaic integration, and label transfer anchored by either histology or omics. (b) For each input slide with spatial multi‐modal data, stMixer employs a multi‐view graph self‐supervision module to learn harmony‐preserving yet modality‐specific representations (top), followed by a self‐looped cross‐attention mechanism that iteratively fuses these features into a unified embedding *Z_i_^O^* (bottom). (c) Given multiple slide‐specific embeddings as input, stMixer jointly optimizes triplet and MMD losses to position biologically similar cells nearby within a shared latent space. (d) For label transfer, stMixer introduces a two‐stage, graph‐enabled cluster‐level voting strategy that propagates annotations from reference profiles to query slides. (e) stMixer enables flexible integration for spatial domain identification and inter‐domain relations characterization, scalable multi‐slide mosaic omics alignment, and cross‐slide label transfer via soft link.

stMixer is a unified framework for mosaic integration and label transfer across multi‐slide spatial histology and multi‐omics datasets (Figure 1a–d). It comprises three core modules: (i) intra‐slide integration (Figure 1b,e), stMixer learns slide‐specific unified embeddings by integrating spatial, molecular, and histological features from an arbitrary number of available modalities. Specifically, modality‐specific and location‐based graphs are jointly modeled through dual‐graph self‐supervision, and the resulting modality representations are adaptively fused by a self‐looped cross‐attention module into a unified embedding *Z* *_i_* *^O^*. This representation supports accurate spatial domain identification and provides the basis for downstream mosaic integration and label transfer; (ii) cross‐slide mosaic integration (Figure 1c,e), stMixer maps slide‐specific embeddings into a shared latent space *Z* by jointly optimizing a triplet loss and an MMD loss. The triplet loss encourages profile‐similar spots or cells across slides to be close while separating dissimilar ones, whereas the MMD loss promotes distributional consistency among analogous clusters. This enables robust and biologically meaningful clustering across tissue sections; and (iii) cross‐slide mosaic label transfer (Figure 1d,e), stMixer combines spatial proximity within the query slide with cross‐slide profile similarity based on histological or omics features. It constructs a query graph and applies cluster‐level soft voting to propagate consensus labels, producing anatomically informed and biologically coherent annotations across mono‐ and multi‐omics slides.

#### 2.1.1 stMixer Supports Scalable Fusion of Arbitrary Multi‐Omics Modalities

**FIGURE 2:**
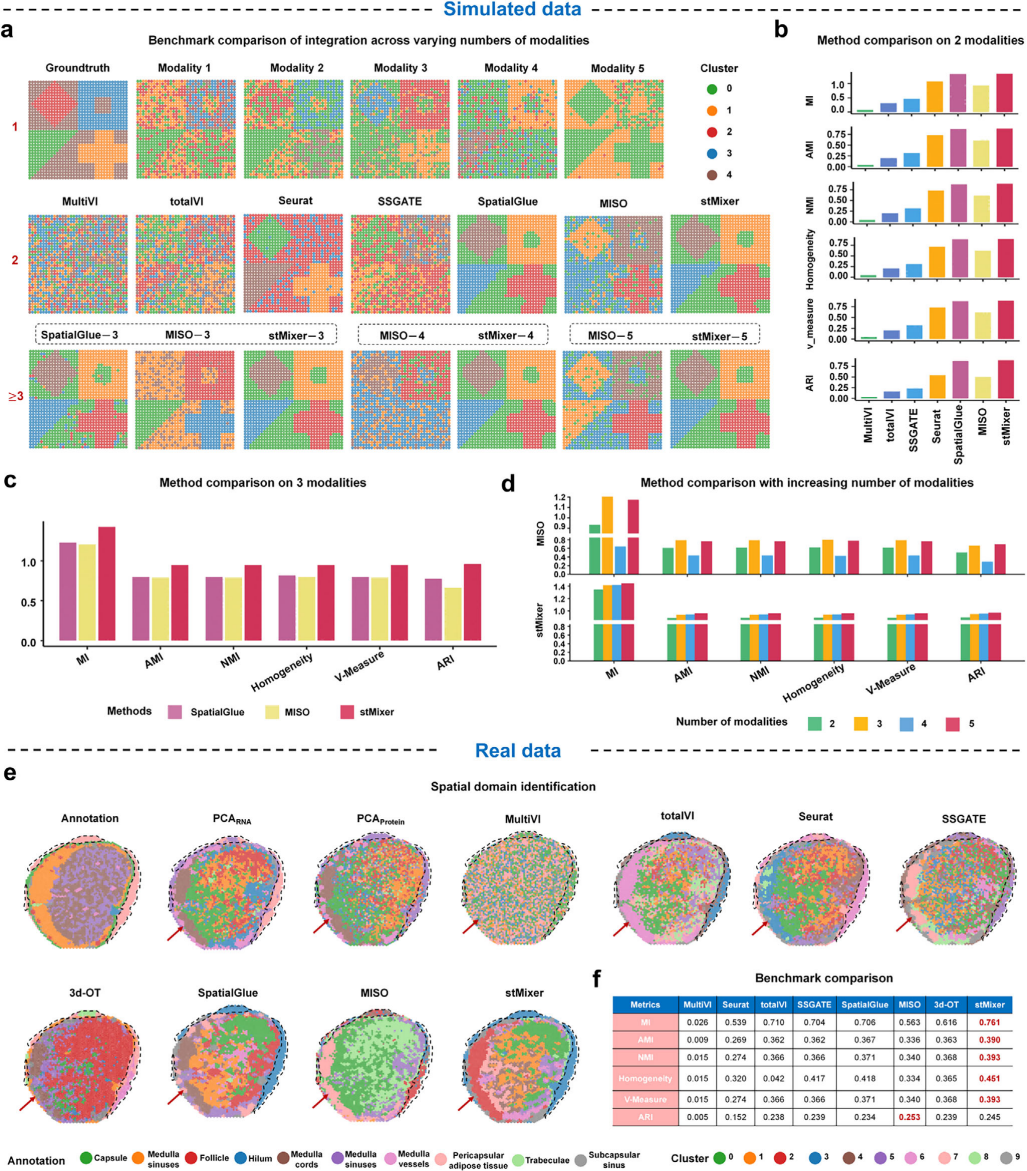
stMixer supports diverse modality integration on both simulated and real datasets. (a) Spatial domain detected from the PCA baseline and integration methods across increasing omics layers (two‐ to five‐omics), shown alongside ground truth, on the simulation dataset. (b) Bar plot comparing seven methods across six clustering metrics for two‐modality integration, on the simulation dataset. (c) Bar plot comparing seven methods across six clustering metrics for three‐modality integration, on the simulation dataset. (d) Bar plot comparing clustering metrics across three methods as the number of integrated modalities increases, on the simulation dataset. (e) Spatial domains identified by eight multi‐omics integration methods, along with a PCA‐based baseline using separate modality clustering, on the human lymph node dataset comprising RNA and protein modalities. Ground‐truth labels are provided for reference. (f) Table showing clustering metrics across eight multi‐omics integration methods on the human lymph node dataset.

To assess the flexibility of stMixer in multi‐omics fusion, we simulated datasets spanning two to five modalities with a shared ground truth [23], sampling each modality from empirically derived distributions (Figure 2a). We benchmarked stMixer against single‐cell integrators (Seurat, MultiVI, and totalVI), spatial methods (SpatialGlue, SSGATE, and MISO), and a PCA‐based baseline involving separate clustering for each modality. Notably, Seurat, MultiVI, totalVI, and SSGATE support up to two modalities, SpatialGlue handles three, while stMixer and MISO scale to any number. Clustering accuracy was evaluated using mutual information (MI), adjusted MI (AMI), and normalized MI (NMI) for label dependency; homogeneity and V‐measure for cluster purity and completeness; and adjusted rand index (ARI) for overall agreement.

By comparison, we found that (i) stMixer's spatial domains most closely match the ground truth across six evaluation metrics (Figure 2a–d); (ii) in two‐modality benchmarks, spatially‐informed tools generally outperform MultiVI and totalVI. Among existing methods, Seurat performs better than SSGATE and MISO, while stMixer achieves a slight improvement over SpatialGlue (Figure 2b); (iii) although UMAP embeddings from totalVI, Seurat, SSGATE, and MISO show clearer separation, stMixer yields the tightest intra‐cluster cohesion (Figure S1a); and (iv) as modalities increase, stMixer's performance steadily improves, whereas MISO and SpatialGlue exhibit declining or unstable results. Together, these results demonstrate that stMixer's cross‐attention architecture offers a robust framework for integrating complementary signals across diverse omics modalities (Figure 2a–d).

We next benchmarked stMixer on 10X Genomics‐derived human lymph node data [16], co‐profiling RNA and protein across a single section annotated into ten anatomical domains: capsule, pericapsular adipose, cortex, medulla sinuses, medulla cords, vessels, etc. (Figure 2e). The annotations served as ground truth for evaluating clustering accuracy. Our analysis revealed that (1) stMixer achieved strong overall performance on this dataset. It obtained an ARI comparable to that of MISO and outperformed the other methods, while also achieving a substantially higher MI. This suggests that stMixer better captures the global correspondence between predicted clusters and anatomical annotations, indicating robust clustering performance and improved preservation of anatomical structure (Figure 2e,f); (2) by jointly leveraging RNA and protein, stMixer delineates structures (e.g., capsule vs. pericapsular adipose) that neither modality alone nor other multi‐omics tools resolve as clearly (Figure 2e). We also compared the full stMixer model with two single‐modality variants, RNA‐only and protein‐only, while keeping the same overall representation learning and integration framework. The full multimodal model achieved the best performance, with an ARI of 0.245, whereas the RNA‐only and protein‐only variants decreased to 0.203 and 0.138, respectively. These results suggest that RNA and protein contribute complementary information to tissue‐domain identification, and that their joint modeling enables more accurate and robust multimodal integration (Figure S2a,b); and (3) Although 3d‐OT recovered part of the spatially continuous structure, regional mixing remained between several domains, such as pericapsular adipose tissue and medulla sinuses, whereas stMixer produced boundaries more consistent with the annotations (Figure 2e). In addition, except for MISO, spatially informed methods generally outperformed pure single‐cell methods, highlighting the importance of spatial information for resolving tissue organization (Figure 2f).

In summary, stMixer scales across diverse modalities to integrate complementary signals into a unified space, enabling accurate and robust spatial domain identification in multi‐omics data.

#### 2.1.2 stMixer Identifies Transited Cell‐States

**FIGURE 3:**
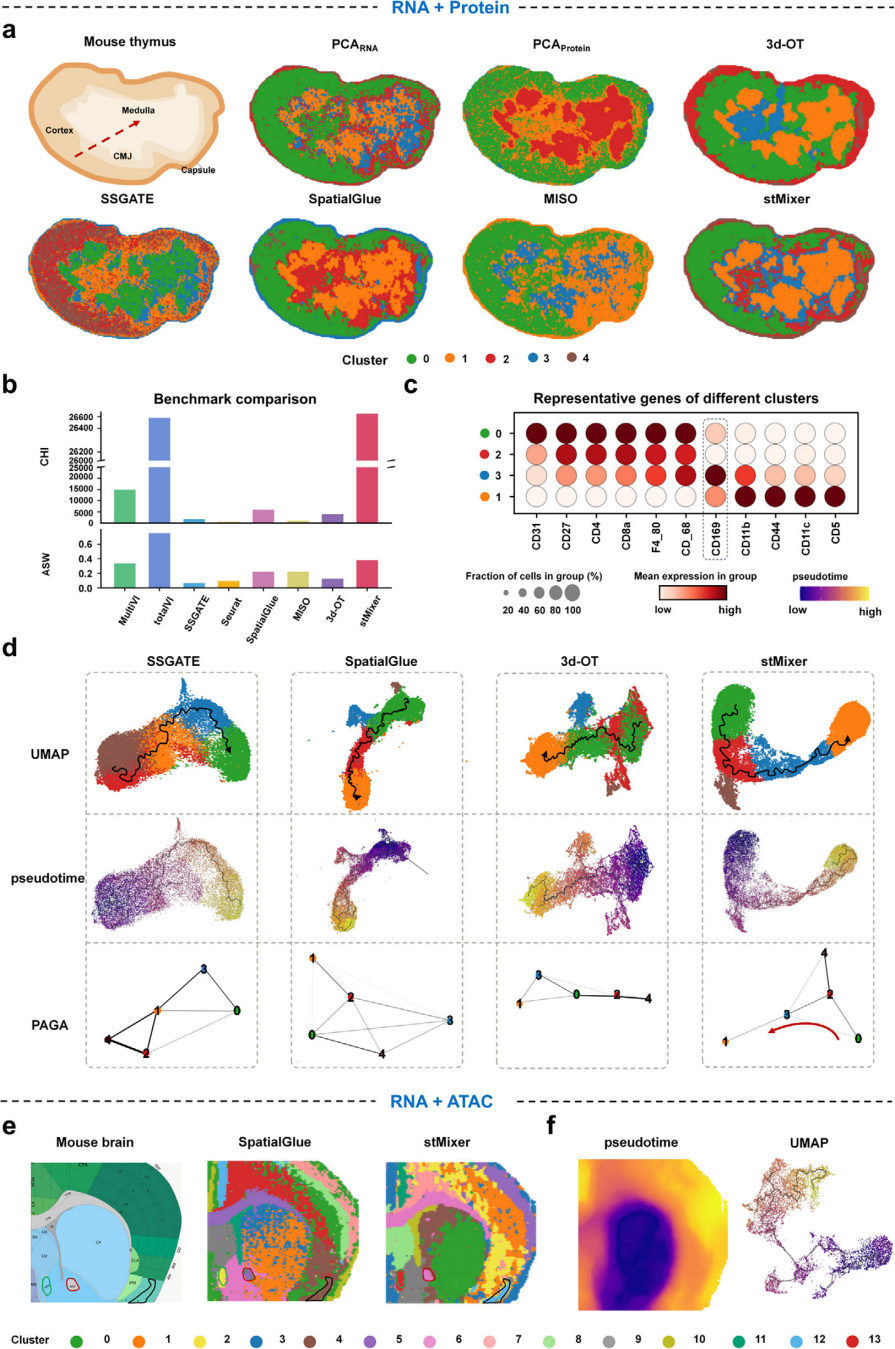
stMixer identifies transitional regions through the integration of spatial multi‐omics data. (a) Spatial domain identified by five multi‐omics integration methods and a PCA‐based baseline (separate clustering per modality) on the mouse thymus dataset, with tissue structure shown for reference. (b) Bar plots comparing the clustering performance of eight methods across two evaluation metrics for RNA–protein integration on the mouse thymus dataset. (c) Dot plot showing protein expression levels of representative markers across four clusters identified by stMixer on the mouse thymus dataset. (d) UMAP embedding (top), pseudo‐time trajectory (middle), and PAGA graph (bottom) inferred by each method, on the mouse thymus dataset. (e) Spatial domains detected by SpatialGlue and stMixer, with reference annotations for comparison on the mouse brain 1 profiled by spatial ATAC–RNA‐seq. (f) Spatial distribution (left) and UMAP embedding (right) of pseudo‐time inferred by stMixer on the mouse brain 1 profiled by spatial ATAC–RNA‐seq.

To further demonstrate the application power of stMixer, we analyzed a mouse thymus dataset profiled using Stereo‐CITE‐seq (RNA and protein) [24] to investigate the relations between distinct spatial domains. The thymus is encapsulated by connective tissue septa into lobules with distinct cortex and medulla regions (Figure 3a and Figure S3a) [25]. We compared stMixer with Seurat, MultiVI, totalVI, SSGATE, MISO, SpatialGlue, 3d‐OT, and independent modality‐specific clustering. Clustering performance was evaluated using the average silhouette width (ASW), which measures intra‐cluster cohesion and inter‐cluster separation, and the Calinski‐Harabasz index (CHI), which quantifies the ratio of between‐cluster to within‐cluster dispersion.

In short, we observed that (i) RNA and protein modalities exhibit distinct yet complementary clustering patterns—protein data delineate a more complete medulla, while RNA data highlight a well‐defined capsule and separate the cortex from the medulla (Figure 3a); (ii) stMixer provides the most effective integration of these complementary patterns, accurately identifying the medulla, cortex, and capsule. SpatialGlue and 3d‐OT also recover these major anatomical structures, but with less distinct boundaries than stMixer, whereas the remaining methods fail to clearly resolve them (Figure 3a and Figure S3b); (iii) although totalVI achieves the highest ASW score, its clustering lacks anatomical precision. In contrast, stMixer achieves the highest CHI score and second‐highest ASW score, indicating more anatomically consistent, well‐separated, and compact clusters (Figure 3b and Figure S3b); and (iv) notably, stMixer uniquely identifies a transitional region (i.e., cluster 3) between the cortex and medulla, corresponding to the cortico‐medullary junction (CMJ), whereas methods including 3d‐OT and SpatialGlue do not clearly resolve this boundary region. Analysis reveals that cluster 3 cells express CD169, a known marker of CMJ‐resident macrophages [26], and the inferred trajectory from cortex to CMJ to medulla recapitulates the known anatomical progression [27], further validating its identity as the CMJ region (Figure 3c,d, and Figure S3c).

Furthermore, to assess generalizability beyond the above two‐modality data, we applied stMixer to the mouse brain dataset generated using the spatial ATAC‐RNA‐seq platform [28]. We noted that stMixer successfully integrates transcriptomic and chromatin accessibility signals, recovering complex structural features, for example, cluster 12, which remains indistinct in the results of other comparators under the original clustering setting (Figure 3e and Figure S4a–c). Notably, the inferred inter‐domain relationships closely match known anatomical structures of the mouse brain (Figure 3f). Cluster 12 is mainly localized to the atlas‐annotated PIR2 region in our sections. Previous studies have shown that the piriform cortex consists of layers I–III, with sparse neurons in layer I, densely packed neurons in layer II, and intermediate neuronal density in layer III [29, 30, 31]. In addition, cluster 12 expressed *Cux1*, which has been reported to be enriched in piriform layers IIb and III [31], and also showed expression of several neuronal and synapse‐related genes, including *Syt1*, *Nrxn2*, and *Map1b* [32, 33, 34] (Figure S4b). Based on this anatomical and molecular evidence, we conservatively interpret cluster 12 as a PIR2‐associated principal‐neuron cluster.

Overall, stMixer delivers precise, coherent spatial multi‐omics integration, capturing complementary signals and resolving transitional regions missed by other methods.

### 2.2 Flexible and Biologically Coherent Integration of Mosaic Spatial Omics

**FIGURE 4:**
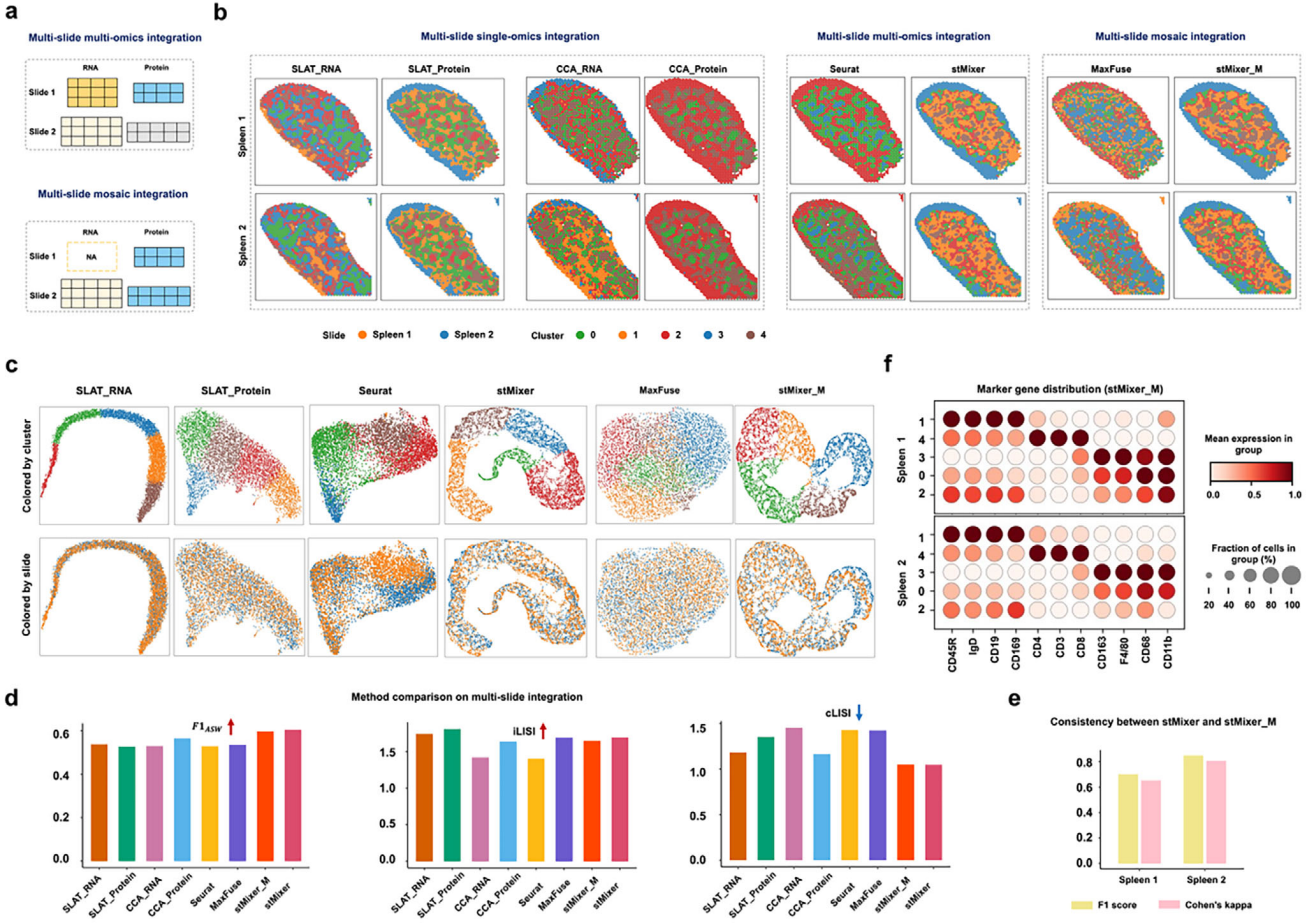
stMixer enables multi‐slide mosaic omics integration on two mouse spleen sections. (a) Schematic illustration of multi‐slide multi‐omics integration and its mosaic variant. (b) Spatial domains identified by single‐omics integration methods (SLAT and Seurat for RNA and protein), multi‐omics methods (Seurat and stMixer), and mosaic integration methods (MaxFuse and stMixer_M). (c) UMAP embeddings generated by six computational methods. (d) Bar plot comparing batch‐effect correction metrics (*F*1_*ASW*_, iLISI, and cLISI) across eight methods. (e) Bar plot showing consistency in cluster predictions between stMixer and stMixer_M across two slides, evaluated by Kappa coefficient and F1 score. (f) Dot plot displaying protein expression levels of 11 representative markers across five clusters identified by stMixer_M.

To assess the capacity of stMixer for multi‐slide soft integration, we applied it to two mouse spleen sections profiled by SPOTS (RNA and protein) [8]. stMixer learns slide‐specific multi‐modal representations, which are then aligned into a shared embedding space through joint optimization of triplet and MMD losses. To further evaluate its flexibility, we simulated a mosaic integration scenario (stMixer_M) by removing RNA from slide 2 and perturbing the protein modality—introducing Gaussian noise to the feature matrix and modifying the multi‐view graph structure to emulate a distinct modality (Figure 4a and Figure S5a,b). In addition to intra‐slide clustering comparisons with Seurat, MultiVI, totalVI, SSGATE, MISO, SpatialGlue, and 3d‐OT, we benchmarked cross‐slide alignment methods such as Seurat and SLAT [35], as well as the mosaic integration method MaxFuse. We used *F*1_*ASW*_, derived from ASW, to assess both cluster separation and slide mixing. The cell‐type local inverse Simpson's Index (cLISI) [36] and integration LISI (iLISI) [37] were further employed to evaluate local purity and integration quality across slides, respectively.

By comparison, we observed that: (i) in single‐slide multi‐modal integration, stMixer achieves the best spatial clustering performance among both single‐cell integrators and spatial methods, as reflected by CHI and ASW scores. Although MultiVI shows slightly higher ASW in spleen 2, its clustering lacks biologically meaningful structures (Figure S6a–c); (ii) in multi‐slide integration, stMixer outperforms all compared methods in balancing inter‐cluster separation and cross‐slide mixing, achieving the highest *F*1_*ASW*_ and superior cLISI, despite SLAT_protein showing a marginally higher iLISI (Figure 4b–d and Figure S7a); (iii) stMixer and its mosaic variant stMixer_M exhibit consistent performance in both slide mixing and spatial domain identification, with high concordance in cluster assignments as measured by Cohen's Kappa and F1 scores (Figure 4d–e), indicating strong robustness and scalability for mosaic integration; and (iv) stMixer, stMixer_M, and SLAT_protein consistently identify three macrophage subtypes within aggregated regions, characterized by distinct marker signatures including CD163, F4/80, CD68, CD11b, and B cell–associated markers. These findings align with previous reports indicating that red pulp macrophages predominantly express F4/80 and CD68 [38, 39], whereas marginal zone macrophages form an inner CD169^+^ layer that interacts with follicular B cells and migrates into follicles upon activation [40, 41, 42] (Figure 4f and Figure S7b); and (v) notably, the cross‐slide multi‐modal integration module—including its mosaic variant—improves macrophage subtype resolution relative to single‐slide multi‐modal integration, as demonstrated by more distinct distributions of marker protein expression (Figure S8); and (vi) additional single‐available‐modality analysis in the mouse spleen dataset showed that either RNA or protein could support cross‐slide soft mosaic integration, with modality‐ and region‐dependent effects (Note S1 and Figure S9).

Collectively, stMixer aligns multi‐slide spatial omics data to enhance region delineation by stronger sections to improve weaker ones, even with missing modalities.

#### 2.2.1 stMixer Supports Unified Label Transfer From Histological or Omics References

**FIGURE 5:**
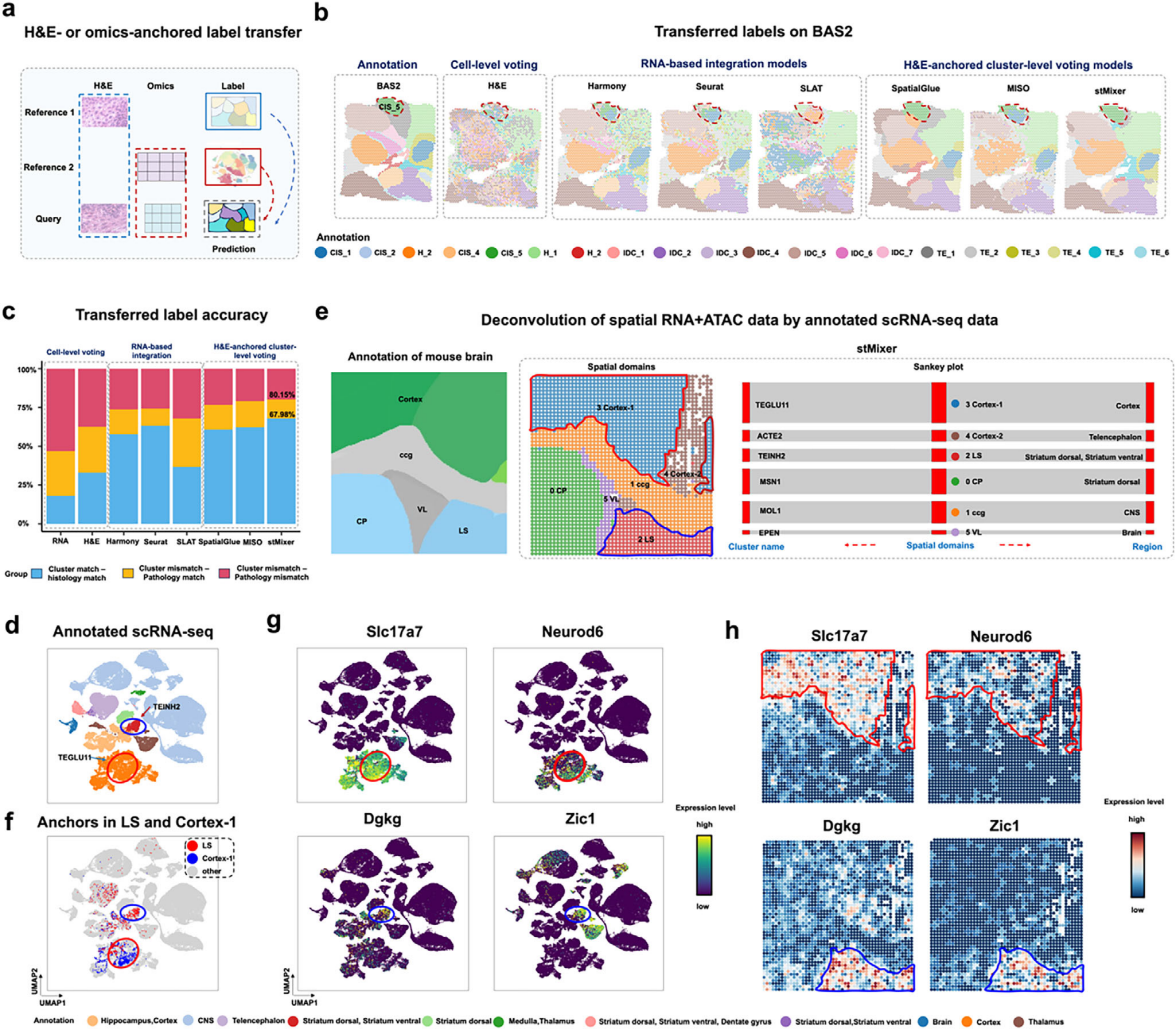
stMixer facilitates multi‐slide mosaic label transfer anchored by histology or omics data. (a) Schematic illustration of cross‐slide label transfer guided by features from either histological images or omics profiles. (b) Transferred spatial domains on the BAS2 slide (human breast cancer) using cell‐level voting methods, RNA‐based integration approaches (Harmony, Seurat, and SLAT), and H&E‐anchored cluster‐level voting methods (SpatialGlue, MISO, and stMixer). Ground‐truth histological annotations are shown for reference. (c) Bar plot comparing label transfer accuracy across eight methods. Bar color indicates whether the evaluation is based on cluster‐level agreement or histological‐type concordance on the BAS2 slide. (d) UMAP embedding of 89 689 cells from the mouse brain scRNA‐seq reference, colored by 11 anatomical regions. (e) Ground‐truth annotations (left) and stMixer‐inferred spatial domains (middle) on mouse brain 2 with spatial RNA‐ATAC profiles, along with a Sankey plot (right) illustrating the correspondence between stMixer predictions, scRNA‐seq‐defined cell types, and anatomical regions (as annotated in the scRNA‐seq dataset). (f) UMAP visualization of anchor cells in the LS and Cotext‐1 regions, inferred by matching on the scRNA‐seq reference. (g) UMAP plots showing the expression patterns of four marker genes (*Slc17a7*, *Neurod6*, *Dgkg*, and *Zic1*) in the scRNA‐seq reference. (h) Spatial expression maps of the same four marker genes (*Slc17a7*, *Neurod6*, *Dgkg*, and *Zic1*) on the mouse brain 2.

To assess histology‐guided label transfer (Figure 5a), we applied stMixer to map annotations from BAS1 to BAS2—two Visium slides of the same human breast cancer tissue, each with H&E, gene expression, and 20 annotated histological regions spanning four histological types (Figure 5b and Figure S10a) [43]. In this label‐transfer setting, H&E and RNA have task‐specific roles. H&E provides the cross‐slide morphological reference for linking histological structures between slides, whereas RNA provides the molecular representation needed for label transfer. We benchmarked stMixer against RNA‐based methods (Harmony, Seurat, and SLAT), histology‐anchored cell‐level voting, and cluster‐level methods (SpatialGlue and MISO). Accuracy was evaluated at both cluster and region levels. Among existing methods, histology‐based cluster voting outperformed RNA‐based and cell‐level strategies. Notably, stMixer further surpasses SpatialGlue and MISO, particularly in delineating the CIS_5 region, highlighting its ability to capture coherent transcriptomic‐histological structures (Figure 5b,c, and Figure S10b).

We further evaluated the versatility of stMixer; we transferred independently derived scRNA‐seq labels [44] onto a spatial‐ATAC‐RNA‐seq‐profiled mouse brain section [28], using RNA as the common anchoring modality (Figure 5d and Figure S11). We found that (1) both stMixer and SpatialGlue more accurately recapitulate known anatomical structures [45] and preserve spatial integrity compared to Seurat and MISO, which fail to do so (Figure 5e and Figure S12); (2) stMixer distinguishes two adjacent cortex clusters: cluster 0, dominated by TEGLU10 and TEGLU11 cells located in the cortex, and cluster 4, enriched for ACTE2 and ACTE1 cells in the telencephalon. This distinction is supported by elevated expression of Slc17a7 and Neurod6 in TEGLU10/11 cells within cluster 0, reinforcing their molecular separation from telencephalic subtypes [46, 47] (Figure 5e–h); and (3) in the LS region, stMixer predicts the label “Striatum dorsal, Striatum ventral”—a region that is anatomically adjacent but functionally distinct from LS. However, elevated expression of LS‐specific marker genes *Dgkg* and *Zic1* in the corresponding scRNA‐seq cells supports their true origin in the LS. This suggests that, in the absence of spatial context, these cells may have been previously misclassified as part of the striatum (Figure 5e–h). Likewise, the marker‐gene expression patterns for the CP, ccg, and VL regions align precisely with the corresponding cell type predictions from the scRNA‐seq data [44] (Figure S13).

Together, these findings highlight stMixer's capacity to resolve subtle anatomical boundaries—crucial for accurate label transfer in complex brain regions.

#### 2.2.2 stMixer Refines Spatial Domain Identification via Cross‐Slide Knowledge Transfer

To assess cross‐slide mosaic label transfer, we applied stMixer to two spatial‐ATAC‐RNA‐seq‐profiled mouse brain sections [28], using intra‐slide representations and Leiden clustering to define reference domains from the multi‐omics slide (Figure S14a). SpatialGlue was excluded due to its lack of single‐modality support, while MISO served as a cluster‐level voting baseline. For comparison, we included leading RNA‐based methods—Harmony, Seurat, and SLAT—using spatial RNA‐seq–derived labels as the reference. stMixer outperformed all benchmarks, accurately resolving five distinct brain regions (Cortex, ccg, CP, VL, and LS), including two cortex subregions validated by canonical markers (Figure S14b,c). Harmony ranked second but failed to resolve the LS and Cortex regions (Figure S14b). Notably, when transferring labels from RNA‐only to multi‐omics data, stMixer was the only approach to recover all major brain regions with coherent spatial localization (Figure S14d,e).

## 3 Discussion

stMixer is a unified and scalable framework for cross‐section mosaic integration and anatomically informed label transfer across spatial histology and multi‐omics datasets. Unlike existing graph‐based models such as SpatialGlue, which mainly target paired multimodal integration within a single tissue slice, stMixer supports arbitrary omics or H&E modalities both within and across slides, including mosaic settings with missing modalities. Methodologically, stMixer offers three key advances: (i) it combines dual‐graph self‐supervised learning with self‐looped cross‐attention to preserve modality‐specific and spatial structures while adaptively fusing multiple modalities into a unified latent representation; (ii) it uses metric learning to align biologically similar cells or spots across sections, enabling coherent and memory‐efficient cross‐slide clustering; and (iii) it introduces a two‐stage graph‐guided soft voting strategy that integrates spatial continuity with cross‐slide profile similarity for anatomically faithful label transfer.

We benchmarked stMixer against existing methods on nine spatial multi‐omics datasets spanning RNA+ATAC, RNA+protein, and RNA+H&E combinations, as well as simulated profiles encompassing up to five synthetic modalities with distinct statistical characteristics. Across these diverse modalities, stMixer consistently outperforms competing approaches in domain identification, mosaic alignment, and label transfer. Its performance scales with modality complexity, demonstrating the strength of intelligent cross‐modal fusion. This was further supported by modality‐specific analyses in the human lymph node RNA+protein and mouse brain RNA+ATAC datasets, where the full multimodal model outperformed single‐modality variants (Note S2 and Figure S2). Additional clustering‐sensitivity analysis in the mouse brain dataset confirmed that the PIR2‐associated structure was not specific to the selected cluster number (Note S3 and Figure S15). In the mouse thymus, stMixer uncovers a transient cortico‐medullary state missed by prior tools, revealing anatomically continuous transitions. Notably, it maintains comparable accuracy across multi‐slide datasets with either shared or partially missing modalities, underscoring its robustness to modality dropout. When transferring labels from unpaired scRNA‐seq data, stMixer resolves two distinct cortical regions with divergent cell type compositions, and correctly reassigns cells misclassified as “Striatum dorsal and Striatum ventral” to the adjacent but functionally distinct LS—highlighting its precision in anatomical boundary refinement and annotation correction.

In addition, we benchmarked the runtime and memory usage of stMixer on simulated datasets. Overall, stMixer showed computational costs comparable to most baseline methods, including SpatialGlue, MISO, and 3d‐OT, remaining within the same order of magnitude across all settings. Although stMixer was not the most efficient method in every case, its computational overhead scaled smoothly with increasing data size and did not introduce disproportionate additional costs. In contrast, SSGATE exhibited substantially higher runtime and memory usage, indicating less favorable scalability (Figure S16).

Looking forward, stMixer's modular architecture is well‐suited to incorporate emerging modalities such as spatial metabolomics and imaging mass spectrometry, and to scale toward organ‐wide, cross‐species, and longitudinal atlases. By enabling high‐resolution, multi‐modal tissue mapping at scale, stMixer lays the foundation for new insights into development, disease progression, and therapeutic response.

## 4 Method

### 4.1 stMixer Model

The stMixer model comprises three core components (Figure 1a–e): (1) *intra‐slide integration module*—a self‐looped cross‐attention mechanism that fuses spatial multi‐omics or H&E features into a unified representation; (2) *inter‐slide alignment module*—a multi‐modal metric learning strategy that combines triplet loss for discriminative alignment with MMD loss for distribution consistency, enabling shared feature learning across slides with heterogeneous modalities; (3) *label transfer module*—a graph‐based, two‐stage cluster‐level voting algorithm that propagates annotations bidirectionally between reference spatial omics/H&E/single‐cell datasets and spatial multi‐omics data (and vice versa for mono‐omics contexts). Ablation studies confirm the importance of these key components, including dual‐graph self‐supervision, self‐looped cross‐attention, triplet loss, and MMD loss (Figures S17 and S18).

### 4.2 Intra‐Slide Integration Module

stMixer learns a unified representation for each slide, ${Z_{i}}^{O}\in R^{d\times n_{i}}$ by integrating spatially and molecularly coherent yet modality‐specific features through a self‐looped cross attention mechanism, where *d* denotes the feature dimension and *n_i_* denotes the number of cells in the slide *i* (Figure 1b). Specifically,

#### 4.2.1 Learning Harmony‐Preserving yet Diverse Representations via Multi‐Graph Self‐Supervision

For each slide *i* and modality *j*, such as omics or H&E, stMixer uses a dual‐graph self‐supervision module to jointly capture both spatial and modality‐specific relationships. Specifically, it constructs two graphs with the same set of *n_i_* cells as nodes: a spatial graph *G*_*i*,*j*_^1^, defined by Euclidean distances between spatial coordinates $S_{i}\in R^{n_{i}\times 2}$, and modality graph *G*_*i*,*j*_^2^, defined by cosine similarity between modality features $X_{i,j}\in R^{n_{i}\times c_{i,j}}$, where *c*_*i*,*j*_ denotes the feature dimensionality of modality *j* on slide *i*. The sensitive analysis results showed that the number of neighbors is four (Figure S19a,b).

For each graph *G*_*i*,*j*_^*m*^ (m∈{1,2}), a graph neural network (GNN) aggregates local neighborhood information via its adjacency matrix ${A_{i,j}}^{m}\in R^{n_{i}\times n_{i}}$ and feature matrix *X*_*i*,*j*_, while a Transformer captures global dependencies across all cells/spots. These components yield 50‐dimensional spatial‐aware embeddings *Z_M_* ^*i*,*j*^ and modality‐aware embeddings *Z_F_* ^*i*,*j*^. To align these two views, stMixer minimizes the mean square error between *Z_M_* ^*i*,*j*^ and *Z_F_* ^*i*,*j*^ as a self‐supervised objective:

(1) $${L_{Mse}}^{i,j}=∥{Z_{M}}^{i,j}-{Z_{F}}^{i,j}{∥}_{2}\;$$

To learn modality‐specific yet mutually aligned representations (e.g., *Z_M_* ^*i*,*j*^ for the *jth* modality of slide *i*), stMixer projects features from all *K* modalities into a common latent space and applies a contrastive loss across each of the $\frac{K(K-1)}{2}$ modality pairs. For any two modalities *p* and *q*, the contrastive loss is defined as follows:

(2)

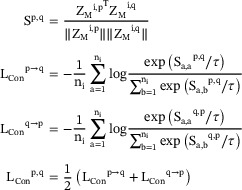

where τ is the temperature parameter, set to 1 by default, and *S*_*a*,*b*_^*p*,*q*^ denotes the cross‐modality cosine similarity matrix between presentations from modalities *p* and *q*. Each element in *S* ^*p*,*q*^ measures the similarity between cells *a* in modality *p* and cell *b* in modality *q*. Thus, the diagonal terms *S*_*a*,*a*_^*p*,*q*^ correspond to matched positive pairs of the same cell across modalities, whereas the off‐diagonal terms *S*_*a*,*b*_^*p*,*q*^ with a≠b serve as negative pairs. The overall loss function for the modality‐specific representation module is therefore:

(3) $$L_{1}=\alpha \sum_{p=1}^{K}{L_{Mse}}^{p,q}+\beta \sum_{1\leq p\leq q\leq K}{L_{Con}}^{p,q}$$

where α and β are used to balance the two loss terms, with default values of 4 and 0.1. We further performed a sensitivity analysis by fixing β  =  0.1 and varying α from 1 to 10, which confirmed that α  =  4 provided the best overall trade‐off between molecular feature preservation and cross‐modal alignment (Figure S19e,f).

#### 4.2.2 Encoding Unified Representations Through Self‐Looped Cross‐Attention

The self‐looped cross‐attention module uses a learnable query as a shared anchor to sequentially integrate modality‐specific representations. For each slide *i*, stMixer initializes a learnable query ${Q_{i}}^{1}=Z_{0}\;\in R^{n_{i}\times 50}$, sampled from a Gaussian distribution *N*(μ, σ). At iteration *j* (1 ≤ *j* ≤ *K*), where *K* denotes the number of available modalities, the spatial‐aware representation of modality *j*, *Z_M_* ^*i*,*j*^, is linearly projected into a key matrix *K_i_* ^*j*^ and a value matrix *V_i_* ^*j*^. The current query *Q_i_* ^*j*^ then attends to *K_i_* ^*j*^ and *V_i_* ^*j*^ through a multi‐head cross‐attention layer to extract modality‐specific information. The attended feature is added back to the query through a residual connection followed by layer normalization, forming a self‐update step. Through *K* iterative rounds, the query progressively accumulates information from all modalities and is refined into a unified query *Q_i_* ^*K* + 1^, thereby capturing cross‐modal semantic information:

(4)

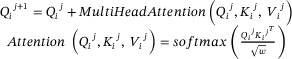




Subsequently, a linear normalization is applied to the final query *Q_i_* ^*K* + 1^ to obtain the joint representation *Z_i_* °. To reconstruct modality‐specific features for modality lll, stMixer further applies a two‐layer linear decoder to *Z_i_* ° as follows:

(5)

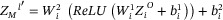

where $W_{i}^{1}$, $W_{i}^{2}$, $b_{i}^{1}$, and $b_{i}^{2}$ are learnable parameters. The reconstruction loss between the original and reconstructed *lth* modality‐specific features is defined as:
(6)

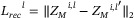




Hence, the total loss function for the intra‐slide multi‐modal integration module is given by:

(7) $$L_{2}=\sum_{l=1}^{K}{L_{rec}}^{l}\;$$

stMixer is trained using the Adam optimizer with a learning rate of 0.001 and a random seed of 10. The dual‐graph self‐supervision module is trained for 100 epochs to learn spatially coherent and modality‐specific representations. The self‐looped cross‐attention module is then trained for 30 epochs to generate the final unified representation.

### 4.3 Cross‐Slide Soft Mosaic Integration Module

stMixer uses metric learning to align slide‐specific representations by optimizing two complementary objectives. Specifically,

- Discriminative alignment with triplet loss: The Leiden algorithm defines intra‐slide clusters. Mutual nearest neighbors (MNNs) based on shared omics or H&E features across slides link cells in identical clusters. Cells with valid MNNs are treated as anchors for training. For each anchor *a*, positives *p* are same‐cluster peers (intra‐slide or MNN across slides), and negatives *n* are cells in other clusters (within‐slide or cross‐slide); a three‐layer MLP projects slide representations into a common space *Z*, the optimization for anchor *a* is defined as:
(8) $$L_{Tri}=\frac{1}{N_{Tri}}\;\sum_{\left(a,p,n\right)\in C_{Tri}}^{N_{Tri}}\max\left(∥Z_{a}-Z_{p}{∥}_{2}-∥Z_{a}-Z_{n}{∥}_{2}+\tau ,\;0\right)$$

where *C_Tri_* represents the set of constructed triplets with a total count *N_Tri_*; and τ is a margin hyperparameter that enforces a minimum separation between positive and negative pairs in the embedding space, and the default value of τ is set to 1.0; and

- Distributional alignment with MMD loss: After 500 triplet‐loss iterations, the shared MLP is further optimized to minimize MMD between embeddings of cells that belong to the same Leiden cluster but originate from different slides (clusters defined by learned embeddings by step i). The squared MMD between two sets of embeddings of cells in cluster *c* across slides *i* and *j* can be written as:
(9) $$\begin{array}{c} \;L_{MMD}=\frac{1}{{n_{i,c}}^{2}}\;\sum_{e,f\in i,c}k\left({Z_{e}}^{O},{Z_{f}}^{O}\right)+\frac{1}{{n_{j,c}}^{2}}\sum_{g,h\in j,c}k\left({Z_{g}}^{O},{Z_{h}}^{O}\right)\\ -\frac{1}{n_{i,c}n_{j,c}}\sum_{\begin{array}{c} e\in i,c\\ g\in j,c \end{array}}k\left({Z_{e}}^{O},{Z_{g}}^{O}\right)\\ k\;\left(u,v\right)=\;\exp\left(-∥u-v{∥}^{2}\right) \end{array}$$

where *n*_*i*,*c*_ and *n*_*j*,*c*_ are the numbers of cells in cluster *c* on sides *i* and *j*. By combining these losses, stMixer ensures that biologically related cells co‐embed tightly across slides while preserving global distributional consistency. In our experiments, subsampling 50% of cells per cluster yields performance comparable to using all cells (Figure S20).

For cross‐slide soft mosaic integration, cross‐slide anchor links are first constructed using reciprocal top‐10 MNNs in the shared‐modality feature space. The alignment module is then trained for 1500 epochs using the Adam optimizer with a learning rate of 1  ×  10^−3^. The triplet margin is set to 1.0.

### 4.4 Cross‐Slide Soft Mosaic Label Transfer Module

To enable robust label transfer from annotated reference datasets (spatial/single‐cell omics or H&E images) to query slides, stMixer uses a two‐stage, graph‐based cluster‐level voting scheme. Specifically,

- Domain‐Level Voting: An unsupervised clustering algorithm first partitions the query slide into initial query domains. In the default implementation, we construct a cosine‐neighbor graph on the query slide with *n_neighbors_* =  15, followed by Leiden clustering to define the query domain *D_d_*. For image‐based label transfer, H&E embeddings are first projected by PCA and corrected by Harmony, and $n_{pca\_he}=20$ components are retained as the common feature space. In this space, each query spot searches for its top‐k cross‐slide reference neighbors, with the default value *k_voting_* =  5, and a majority vote assigns each domain the label:
(10) $$\begin{array}{c} \hat{y}\;\left(D_{d}\right)=arg\max_{c\in C_{r}}\sum_{a\in N_{k}\left(D_{d}\right)}I\left(\;y_{a}=\;c\right)\\ N_{k}\;\left(D_{d}\right)=\underset{u\in D_{d}}{\cup }N_{k}\left(u\right)\; \end{array}$$

where I is the indicator function, *C_r_* denotes the set of annotated reference labels, *y_a_* is the known label of the reference spot or cell *a*, and *N_k_* (*D_d_*) denotes the set of top‐k reference neighbors linked to query spots *u* in domain *D_d_* by cross‐slide neighbor links; and

- Component‐Level Refinement: Because a cluster‐level prediction may contain spatially disconnected regions, stMixer further refines each predicted query domain using the spatial coordinates of the query slide. For each query domain *D_d_*, let *V_d_* denote the set of query cells or spots in this domain. Here, *u* and *v* are query spots within the same query domain *D_d_* and their spatial coordinates are denoted as *s_u_* and *s_v_*. We construct a radius‐based spatial proximity graph:
(11) $$\begin{array}{c} G_{d}=\left(V_{d},E_{d}\right)\;,\;\;E_{d}=\left\{\left(u,v\right)\in V_{d}\times V_{d}\;|\;u\neq v,\;∥s_{u}-s_{v}{∥}_{2}\leq R\right\} \end{array}$$

where the default radius is *R*  =  1000 *pixels* in the full‐resolution image coordinate system; in the human breast cancer Visium dataset, this corresponds to approximately 310 µ*m*, which is about three Visium spot‐center spacings. The connected components of *G_d_* are then identified as spatially contiguous sub‐domains $\left\{S_{1},\text{…},S_{m}\right\}$. Each sub‐domain is relabeled by re‐voting based on its local reference‐neighbor label distribution, and regions lacking any links to the reference are designated as query‐specific niches. This two‐stage approach leverages both transcriptomic similarity and spatial topology to propagate labels in a biologically coherent manner across heterogeneous slides.

As a representative robustness check, we further evaluated the sensitivity to *k_voting_* in Figure S19c,d. The results show that cluster‐level transfer performance remains stable around the default setting.

### 4.5 Datasets and Preprocessing

#### 4.5.1 Simulated Spatial Omics Dataset

We simulated spatial multi‐omics data using the NSF simulation framework [23], generating 1296 cells with five simulated modalities, each modeled with modality‐specific distributions—including zero‐inflated negative binomial for modality 1, Gaussian for modality 2, negative binomial for modality 3, log‐normal for modality 4, and Bernoulli for modality 5—to reflect their distinct statistical properties. All modalities share the same ground‐truth cluster labels, enabling direct evaluation of integration performance.

#### 4.5.2 Real Spatial Omics Dataset

In our study, we utilized nine publicly available datasets across diverse spatial omics technologies in mouse and human tissues. Specifically, (i) Human lymph node: a spatial RNA–protein dataset derived from a formalin‐fixed, paraffin‐embedded (FFPE) human lymph node, profiled using the 10X Genomics platform [16]. The dataset contains 3484 spots, 18 085 genes, 31 protein markers, and 10 annotated clusters; (ii) Mouse thymus: a spatial RNA–protein dataset derived from mouse thymus tissue, generated using the Stereo‐CITE‐seq technology [24]. A total of 20125 spatial barcoded cells were captured, encompassing 26 439 genes and 216 protein markers; (iii) Mouse brain: three spatial RNA‐ATAC datasets from mouse brain tissue were profiled using the spatial ATAC‐RNA‐seq platform [28]. Mouse brain 1 (Figure 3), mouse brain 2 (Figure 5), and mouse brain 3 (Figure S14) comprise 9215, 2497, and 2372 cells, respectively, with each data containing 18 107 genes and 161 514 chromatin accessibility peaks; (iv) Mouse spleen: two spatial RNA–protein datasets derived from mouse spleen tissues, generated using the SPOTS platform [8]. The two replicates contain 2653 and 2768 cells, respectively, and collectively encompass 32 285 genes and 21 protein markers; and (v) Human breast cancer: two spatial transcriptomics slides (BAS1 and BAS2) derived from the same breast cancer tissue, encompassing 3987 and 3789 spots, respectively. Each slide includes gene expression profiles, spatial coordinates, and corresponding H&E images. A total of 20 histologically annotated regions are included to facilitate benchmark comparison [43].

#### 4.5.3 Real scRNA‐seq Dataset

To deconvolute the complex architecture of the mouse brain using stMixer, we leveraged a reference atlas of 89 689 cells spanning 11 anatomical regions and 75 distinct cell types [44].

#### 4.5.4 Preprocessing

To demonstrate the scalability of stMixer, we applied it to integrate diverse omics modalities—including RNA, ATAC, protein, simulated modalities—and H&E data. Specifically, RNA expression and modality 1 data were processed using the standard Scanpy workflow by selecting 3000 highly variable genes and extracting 50 principal components (PCs). ATAC‐seq and modality 3 data were transformed into 50‐dimensional features using latent semantic indexing (LSI). Protein and modalities 2, 4, and 5 data were normalized via log‐ratio transformation followed by PCA, with 50 PCs retained. For modalities with a limited number of features (e.g., protein), the number of retained components was set to the total number of features minus one. Moreover, histological features were extracted using a pre‐trained Vision Transformer (ViT) encoder [48], and subsequently reduced to 50 dimensions via PCA. Notably, the same preprocessing pipeline was applied to both simulated and real datasets.

#### 4.5.5 Benchmark Comparison

For benchmark comparison, we followed each method's original or recommended pipeline whenever possible, as summarized in Table S1. Except for methods that directly produced clustering labels for evaluation, embedding outputs were converted into clustering labels using the same Leiden clustering procedure. The Leiden resolution was dynamically adjusted to predefined target cluster numbers, with details in Note S4 and Table S2. All methods were evaluated using task‐specific metrics.

### 4.6 Evaluation of Clustering

We evaluated clustering performance using a suite of widely adopted metrics [16, 49]. Specifically, MI, AMI, and NMI are used to assess the dependency between predicted clusters and ground‐truth labels; Homogeneity and V‐measure are applied to evaluate cluster purity and completeness; ARI quantifies overall agreement between predicted and true labels; and ASW evaluates the cluster separation by calculating the similarities of features between cells (or spots) within the predicted clusters.

### 4.7 Evaluation of Batch‐Effect Correction

To comprehensively evaluate the performance of batch correction algorithms, we adopted local inverse Simpson's index (LISI) and F1 score [36]. Specifically,

- LISI uses a fixed perplexity to define local neighborhoods, selects nearest neighbors based on local distribution, and computes the inverse Simpson's index to quantify diversity—representing the effective number of distinct labels within each neighborhood. In integration LISI (iLISI), scores are calculated based on batch labels, with higher scores (approaching the expected number of batches) indicating better mixing. In contrast, cell type LISI (cLISI) evaluates biological conservation, where scores close to 1 reflect well‐preserved and biologically pure clusters; and
- *F*1_*ASW*_ score evaluates cluster purity and slide mixing based on ASW. ASW_*slice*_ calculates the ASW value using slide labels as groups, while ASW*_cluster_* determines the ASW value using cluster labels as groups. The specific formula is defined as follows:

(12)

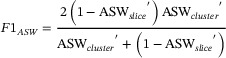

where 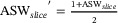, and 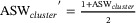. A higher *F*1_*ASW*_ indicates better slide integration.

### 4.8 Agreement Assessment Between stMixer and stMixer_M

To evaluate the consistency between predicted labels from stMixer and its mosaic variants (stMixer_M) in multi‐slide integration, we employed Cohen's Kappa coefficient and macro‐averaged F1 score. Specifically, (1) Cohen's Kappa quantifies inter‐method concordance while adjusting for chance‐level concordance, defined as:

(13) $$K=\frac{p_{o}-p_{e}}{1-p_{e}}\;$$

where *p_o_* denotes the observed agreement, and *p_e_* is the expected agreement under random assignment. The coefficient ranges from −1 (i.e., complete disagreement) to 1 (i.e., perfect agreement), with 0 indicating random‐level concordance; and (2) F1 score quantifies the harmonic mean of precision and recall for each class, offering a balanced measure under class imbalance. It is computed as:

(14) $$\begin{array}{c} F1=2\times \frac{Precision\times Recall}{Precision+Recall}\\ Precision=\frac{TP}{Tp+FP}\\ Recall=\frac{TP}{Tp+FN} \end{array}$$

In multi‐class settings, we report the macro‐averaged F1 score by computing F1 per class and averaging across classes.

## Author Contributions

**Chunman Zuo** conceived and designed the study. **Qixing Yang, Yan Wang,** and Chunman Zuo implemented the model and performed the experiments. Chunman Zuo and Qixing Yang wrote the manuscript with feedback from all authors. **Luonan Chen** and **Yan Wang** analyzed the experimental results. Chunman Zuo, Luonan Chen, and Yan Wang co‐supervised the study. The authors read and approved the final manuscript.

## Conflicts of Interest

The authors declares no conflicts of interests.

## Supporting information




**Supporting File**: advs75905‐sup‐0001‐SuppMat.docx.

## Data Availability

stMixer was applied to both synthetic benchmarks and nine real‐world spatial datasets generated by five different technologies, covering lymph node, thymus, brain, spleen, and breast cancer.The human lymph node dataset was profiled using the 10x Genomics platform and is available from SpatialGlue (Long et al. 2024).The mouse thymus dataset was generated using Stereo‐CITE‐seq (Liao et al. 2023, unpublished), and can be downloaded from the Spatial Transcript Omics DataBase(https://db.cngb.org/stomics/project/STT0000094). Three mouse brain datasets were profiled using the spatial ATAC‐RNA‐seq platform (Zhang et al. 2023), and have been deposited in the Gene Expression Omnibus under accession code GSE205055 (https://www.ncbi.nlm.nih.gov/geo/query/acc.cgi?acc=GSE205055). Two mouse spleen datasets were generated using the SPOTS platform (Ben‐Chetrit et al. 2023), and have been deposited in the Gene Expression Omnibus under accession number GSE198353 (https://www.ncbi.nlm.nih.gov/geo/query/acc.cgi?acc=GSE198353). Two human breast cancer datasets are available on the 10x Genomics website (https://www.10xgenomics.com/datasets/). The mouse brain single‐cell dataset (Zeisel et al. 2018) is deposited in the sequence read archive under accession code SRP135960 (https://www.ncbi.nlm.nih.gov/sra/SRP135960).
