# Supplementary material for: stMixer for Scalable Mosaic Integration and Label Transfer in Spatial Histology and Multi‐Omics
Source: Adv Sci (Weinh). 2026 Jun 2:e75905. Online ahead of print. doi: 10.1002/advs.75905 (PMC13336725; doi:10.1002/advs.75905)
Supplement: Supplementary file 1 — Supporting File: advs75905‐sup‐0001‐SuppMat.docx. [file ADVS-9999-e75905-s001.docx]

**Supplementary information of**

**stMixer for scalable mosaic integration and label transfer in spatial histology and multi-omics**

Qixing Yang^1,2^, Yan Wang^1^*, Luonan Chen^3,4,5^*, Chunman Zuo^2^*

^1^ Key Laboratory of Symbol Computation and Knowledge Engineering of Ministry of Education, College of Computer Science and Technology, Jilin University, Changchun 130012, China

^2^ State Key Laboratory of Biocontrol, Innovation Center for Evolutionary Synthetic Biology, School of Life Sciences, Sun Yat-sen University, Guangzhou 510275, China

^3^ School of Mathematical Sciences and School of AI, Shanghai Jiao Tong University, Shanghai 200240, China

^4^ Key Laboratory of Systems Health Science of Zhejiang Province, School of Life Science, Hangzhou Institute for Advanced Study, University of Chinese Academy of Sciences, Chinese Academy of Sciences, Hangzhou 310024, China

^5^ Tianfu Jincheng Laboratory, Chengdu 610212, China

* To whom correspondence should be addressed.

Email: [wy6868@jlu.edu.cn](mailto:wy6868@jlu.edu.cn); [lnchen@sjtu.edu.cn](mailto:lnchen@sjtu.edu.cn); and [zuochm@mail.sysu.edu.cn](mailto:zuochm@mail.sysu.edu.cn)

**Supplementary Notes**

**Supplementary Note 1. Single-available-modality analysis for cross-slide soft mosaic integration**

We further evaluated whether different modalities can independently serve as the available modality for cross-slide alignment. Using the mouse spleen dataset, we compared two mosaic settings: protein from spleen1 with full multimodal input from spleen2, denoted as stMixer_M(protein), and RNA from spleen1 with full multimodal input from spleen2, denoted as stMixer_M(RNA). Both settings successfully supported cross-slide integration, indicating that either RNA or protein can be used as the available modality for soft mosaic alignment (Supplementary Fig. 9a, b). However, their effects were not identical. stMixer_M(protein) achieved higher F1-ASW and iLISI scores than stMixer_M(RNA), suggesting better batch-effect correction and cross-slide mixing in this dataset, while the cLISI difference was small (Supplementary Fig. 9c). Spatial comparison further supported this modality-specific effect. In cluster 3, stMixer_M(protein) produced a more homogeneous spatial pattern than stMixer_M(RNA), consistent with the PCA baselines, suggesting that protein provides a clearer signal for this region. In contrast, cluster 4 showed similar spatial patterns across RNA- and protein-based settings, indicating that some structures can be recovered by either modality (Supplementary Fig. 9a). Together, these results show that RNA and protein can both support cross-slide soft mosaic integration, but their contributions are modality- and region-dependent.

**Supplementary Note 2. Modality-specific analysis in the mouse brain RNA+ATAC dataset**

We further performed two complementary analyses to evaluate whether the recovery of this structure was supported by complementary modality-specific signals and remained stable across different clustering resolutions. The results showed that RNA and ATAC contributed differently to spatial domain recovery. Cluster 6 was largely recovered by the RNA-only model, whereas cluster 13 was better captured by the ATAC-only model. By contrast, cluster 12 was not fully resolved by either single-modality model but was more clearly delineated by the full RNA+ATAC model (Supplementary Fig. 2c). These results suggest that the two modalities provide complementary information for spatial domain identification.

**Supplementary Note 3. Clustering-sensitivity analysis in the mouse brain dataset**

We examined whether the comparison depends on the selected number of clusters. We re-clustered the fixed embeddings of stMixer, SpatialGlue, and 3d-OT using target cluster numbers $k=10-20$ on the mouse brain RNA+ATAC dataset (Supplementary Fig. 15). The results show that a larger $k$ increases clustering granularity and can introduce local fragmentation. Nevertheless, the putative PIR2-associated region highlighted in Fig. 3e is consistently recovered by stMixer across multiple nearby $k$ values, especially when $k\geq12$. By contrast, at lower $k$ values, 3d-OT fails to distinguish this region from nearby domains, and SpatialGlue shows stronger variation in spatial continuity and boundary definition across $k$. Thus, although cluster number influences spatial-domain resolution, the main comparison in Fig. 3e is not dependent on a single $k=14$ setting.

**Supplementary Note 4. Dataset-specific target cluster numbers for dynamic Leiden clustering**

Leiden clustering resolution does not correspond to a fixed number of clusters, because its effect depends on dataset size, graph structure, embedding distribution, and method-specific representations. Therefore, using a single fixed resolution across datasets or methods may lead to inconsistent clustering granularity.

To ensure comparable granularity, we used a dataset-specific target-cluster-number strategy for all analyses requiring Leiden clustering. For each dataset, the target number of clusters was determined based on the original publication, available histological annotation, or reference annotation. Leiden resolution was then dynamically searched within a predefined range until the target cluster number was reached, with a maximum of 50 iterations.

For method comparison, all methods applied to the same dataset used the same target cluster number. The target numbers and their selection bases are summarized in Supplementary Table 2.

**Supplementary Figures**


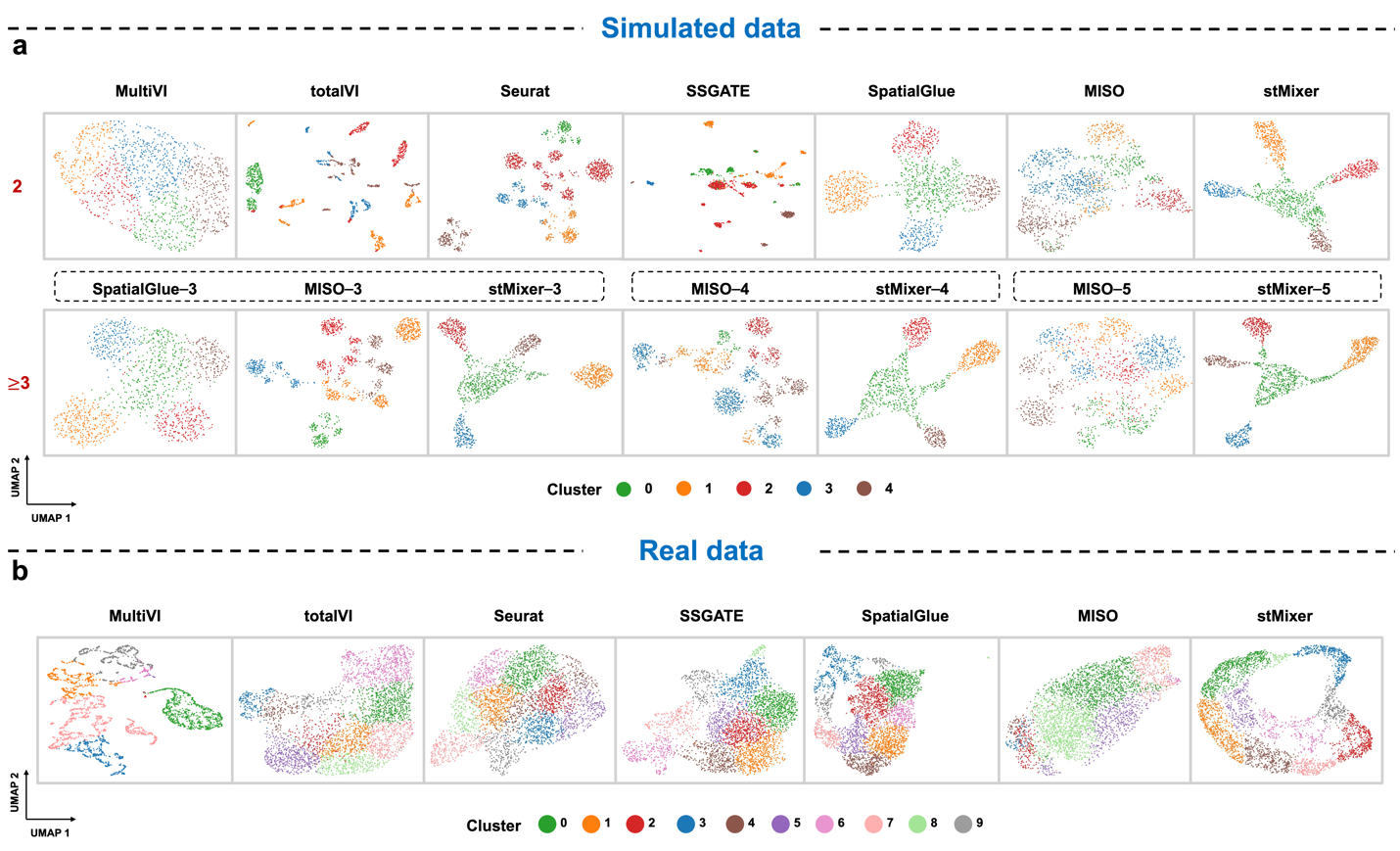


**Supplementary Figure 1**. **Method comparison on simulated and real human lymph node datasets.** **a** UMAP visualization of latent features generated by multi-omics integration methods, including single-cell tools (MultiVI, totalVI, and Seurat) and spatial tools (SSGATE, SpatialGlue, MISO, and stMixer), on the simulation dataset with the number of omics ranging from two to five. **b** UMAP plots of latent features from various integration methods on the human lymph node dataset. In each panel of **a** and **b**, colors represent predicted clusters.


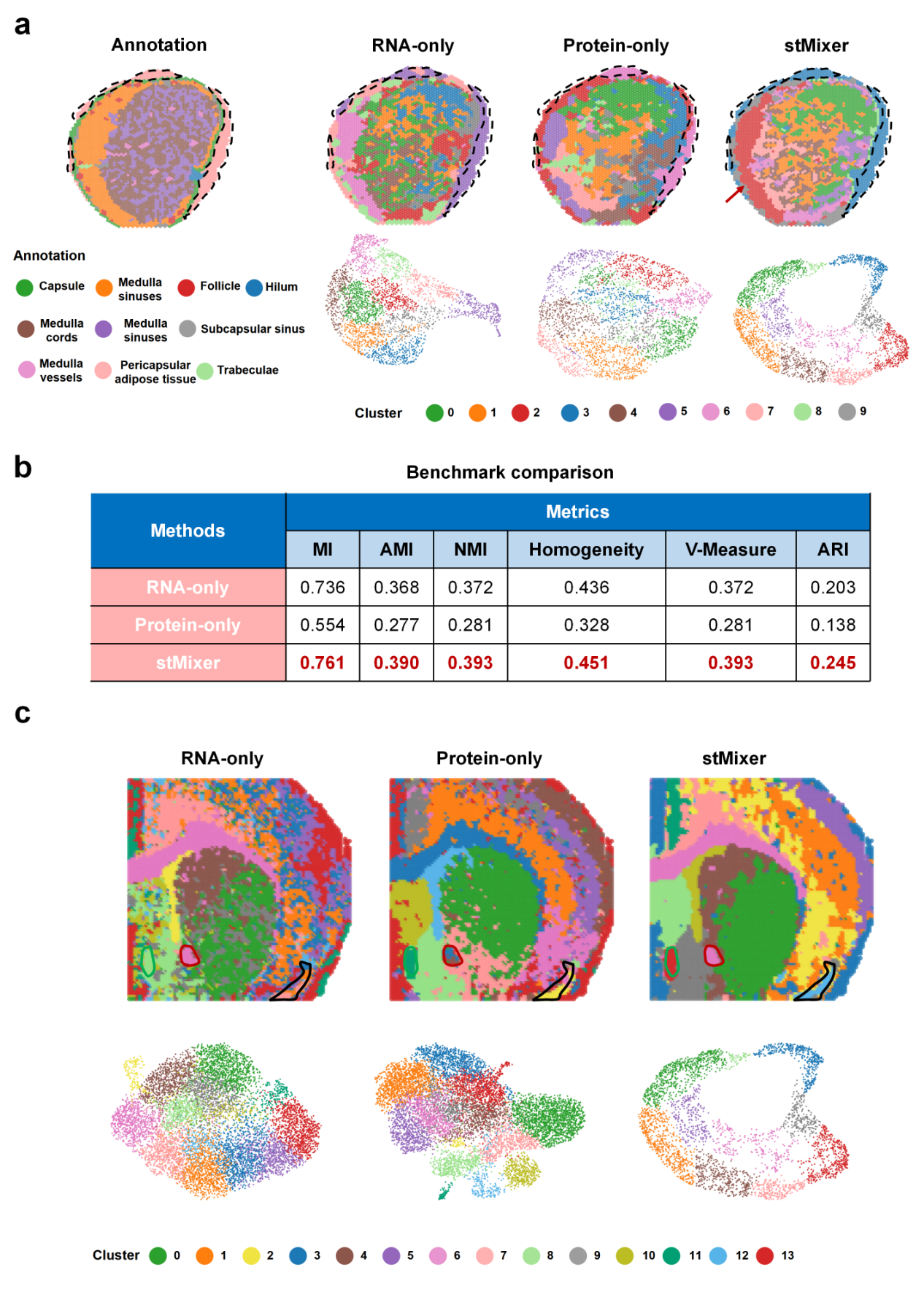


**Supplementary Figure 2. Evaluation of the contributions of different modalities to spatial domain identification on the human lymph node and mouse brain datasets. a** Spatial domains and UMAP embeddings generated by stMixer, stMixer using only the RNA modality as input, and stMixer using only the protein modality as input on the human lymph node dataset. Ground-truth labels are shown for reference. **b** Table summarizing clustering metrics for stMixer, stMixer using only the RNA modality as input, and stMixer using only the protein modality as input. **c** Spatial domains and UMAP embeddings generated by stMixer, stMixer using only the RNA modality as input, and stMixer using only the ATAC modality as input on the mouse brain dataset.


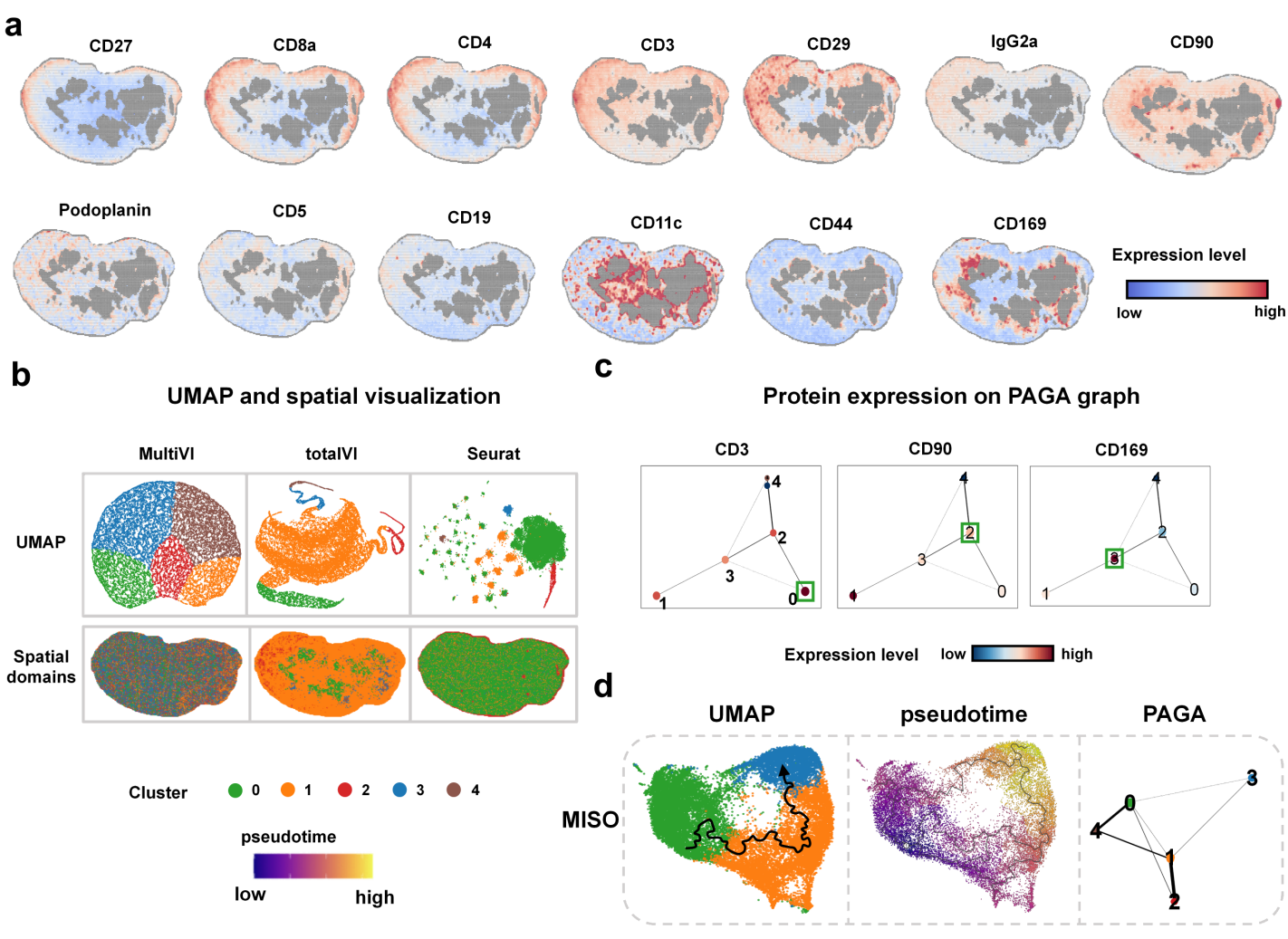


**Supplementary Figure 3**. **Method comparison on the mouse thymus dataset profiled with Stereo-CITE-seq (integrating RNA and protein modalities).** **a** Spatial expression patterns of 13 protein markers. **b** Spatial domains and UMAP visualization of latent features generated by MultiVI, totalVI, and Seurat, with colors indicating predicted clusters. **c** Protein expression levels of three representative markers for three cell types, visualized on the PAGA graph constructed from the low-dimensional features learned by stMixer. **d** UMAP embedding, pseudo-time trajectory, and PAGA graph inferred by MISO, on the mouse thymus dataset.


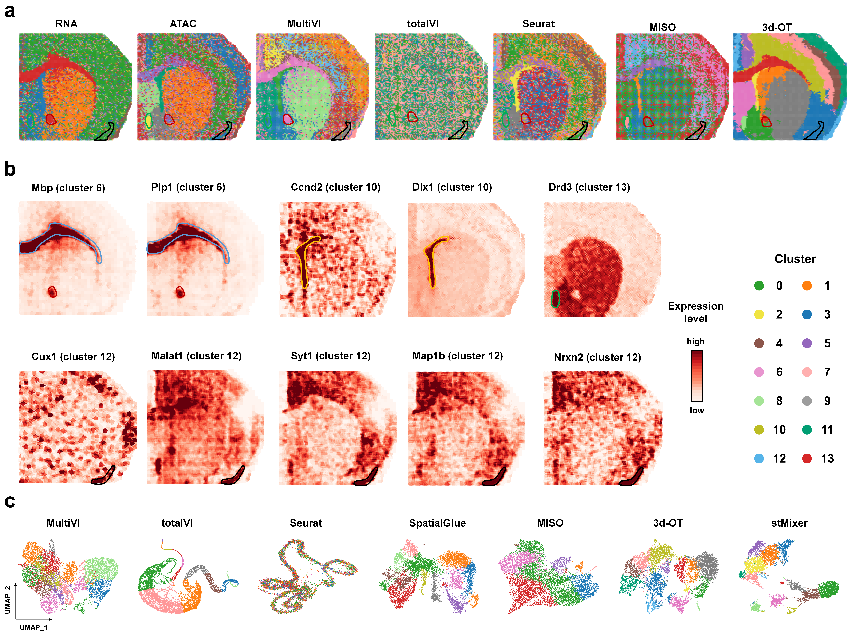


**Supplementary Figure 4**. **Method comparison on the mouse brain 1 profiled using Spatial ATAC-RNA-seq (integrating RNA and ATAC modalities). a** Spatial domain identified by PCA-based dimensionality reduction followed by independent clustering on each modality, and multi-omics integration methods including MultiVI, totalVI, Seurat, MISO, and 3d-OT. **b** Spatial expression patterns of ten markers. **c** UMAP visualization of latent features generated by MultiVI, totalVI, Seurat, SpatialGlue, MISO, 3d-OT, and stMixer, with colors indicating predicted clusters.


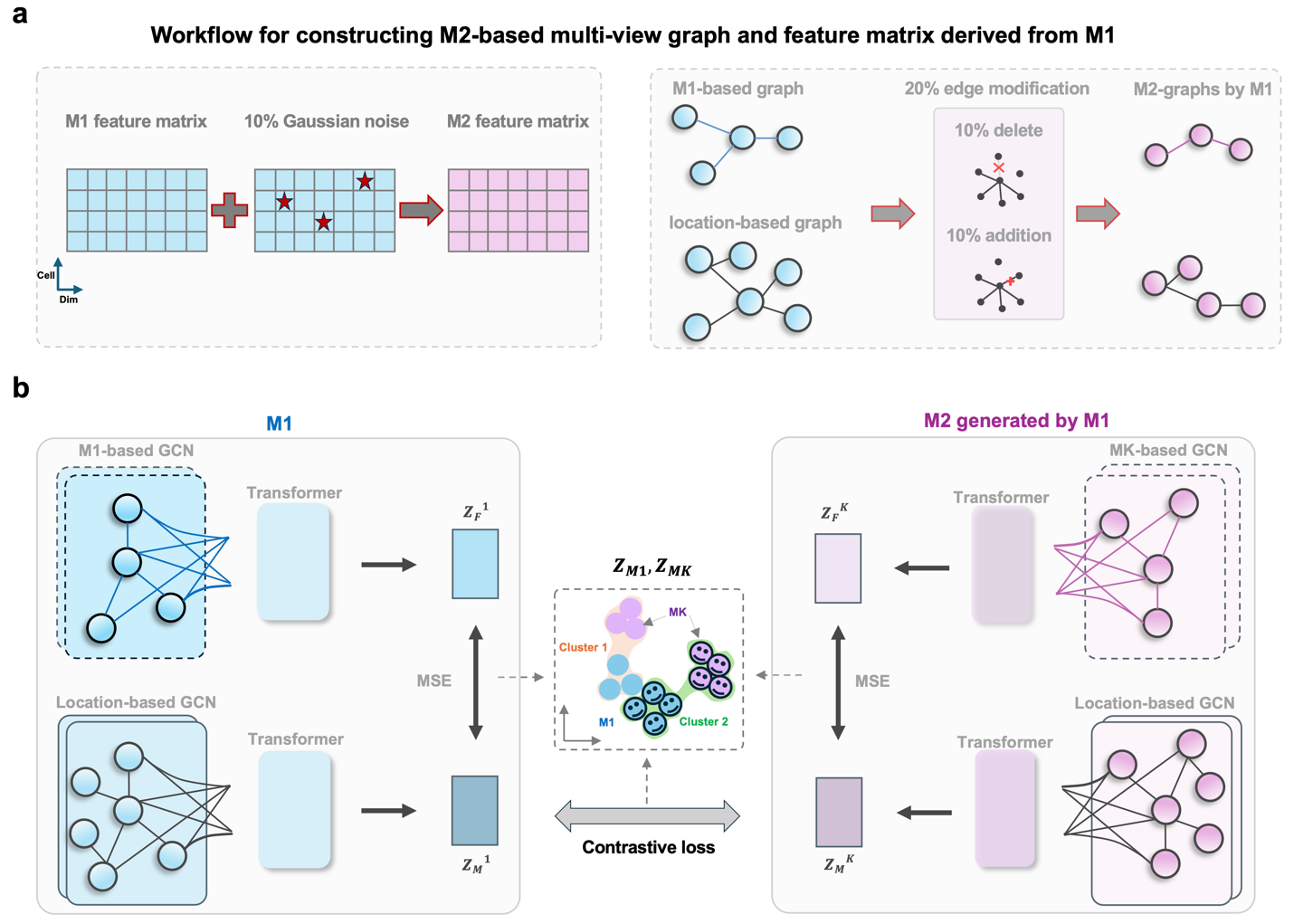


**Supplementary Figure 5**. **Workflow of stMixer for single-modality representation learning. a** Generation of M2-based feature matrix (left) and multi-view graph (right) from M1. **b** Learning modality-specific yet harmony-aligned representations through dual-graph self-supervision.


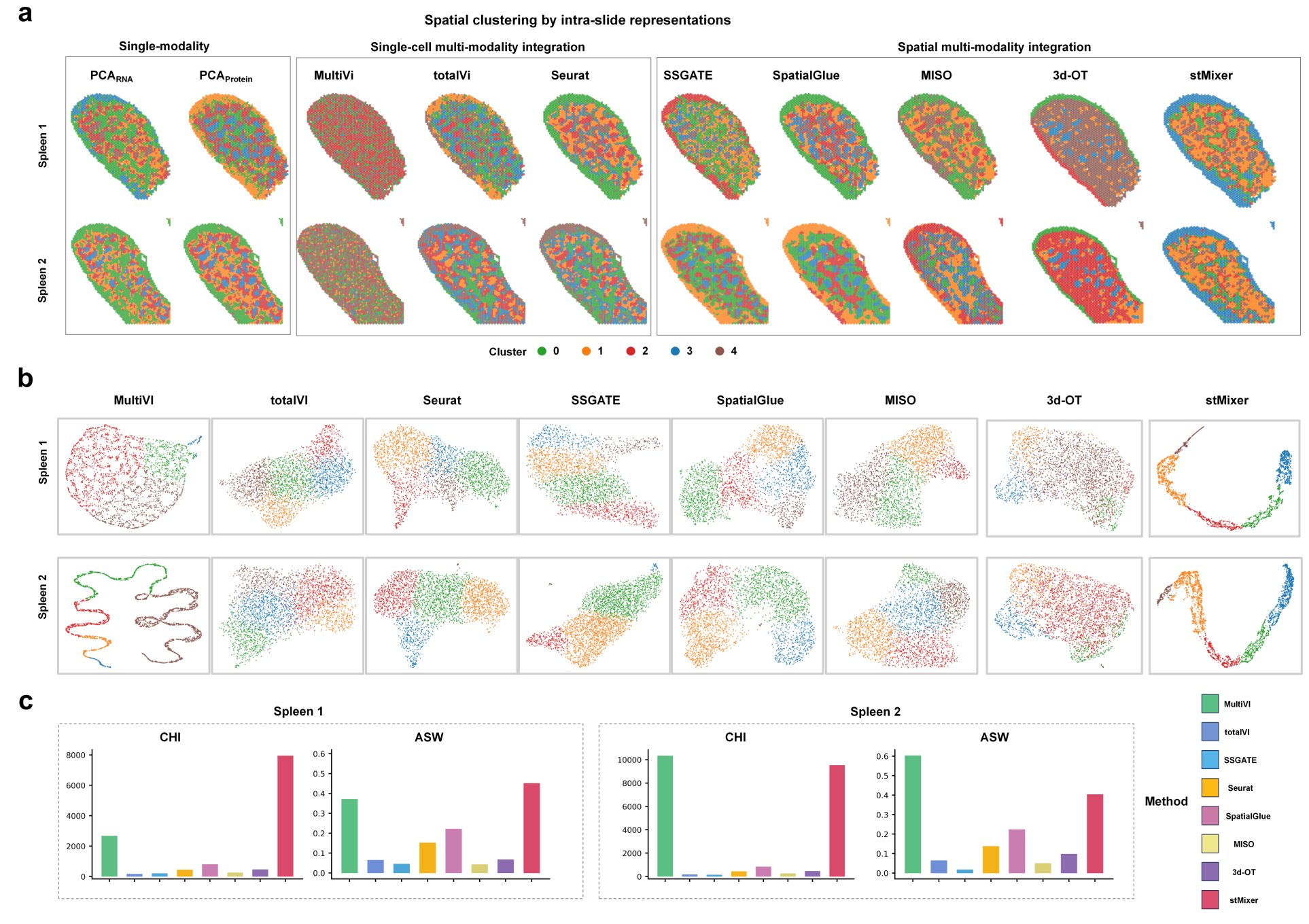


**Supplementary Figure 6**. **Method** **comparison for learning slide-specific representations from RNA and protein profiles in two SPOTS-based mouse spleen tissue sections. a** Spatial domain identified by single-cell tools (MultiVI, totalVI, and Seurat) and spatial tools (SSGATE, SpatialGlue, MISO, 3d-OT, and stMixer), as well as PCA-based dimensionality reduction followed by independent clustering on each omics. Each color indicates one cluster. **b** UMAP visualization of latent features generated by multi-omics integration methods, including single-cell tools (MultiVI, totalVI, and Seurat) and spatial tools (SSGATE, SpatialGlue, MISO, 3d-OT, and stMixer). **c** Quantitative clustering evaluation using calinski-harabasz index (CHI) and average silhouette width (ASW), on two slides.


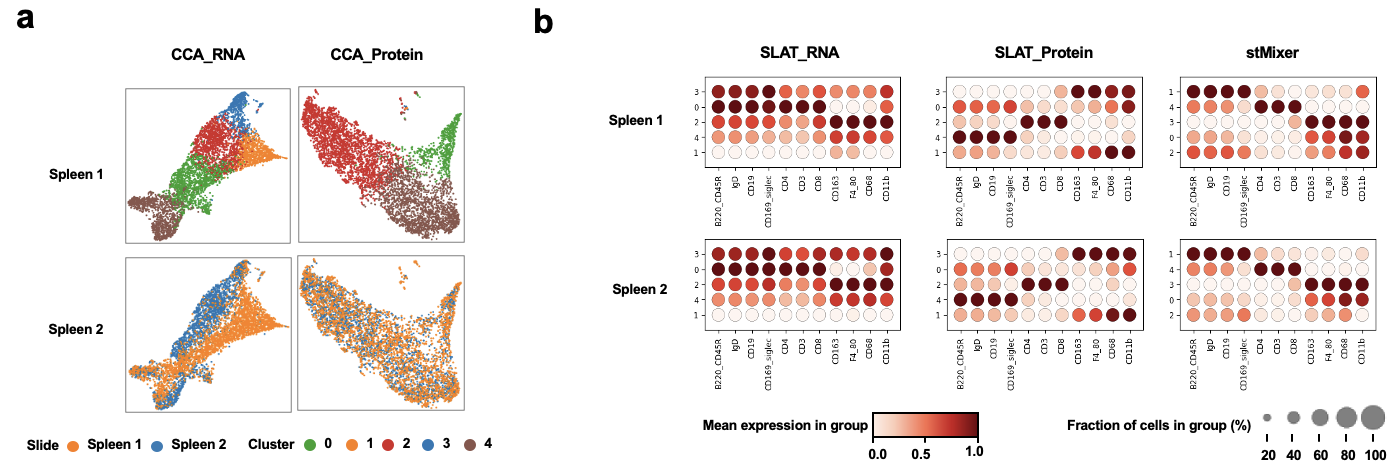


**Supplementary Figure 7. Method comparison for multi-slice integration in two SPOTS-based mouse spleen tissue sections. a** UMAP visualization of latent features generated by CCA on RNA and protein, respectively. **b** Dot plot showing the distribution of representative protein markers across five clusters predicted by SLAT_RNA, SLAT_Protein, and stMixer, respectively.


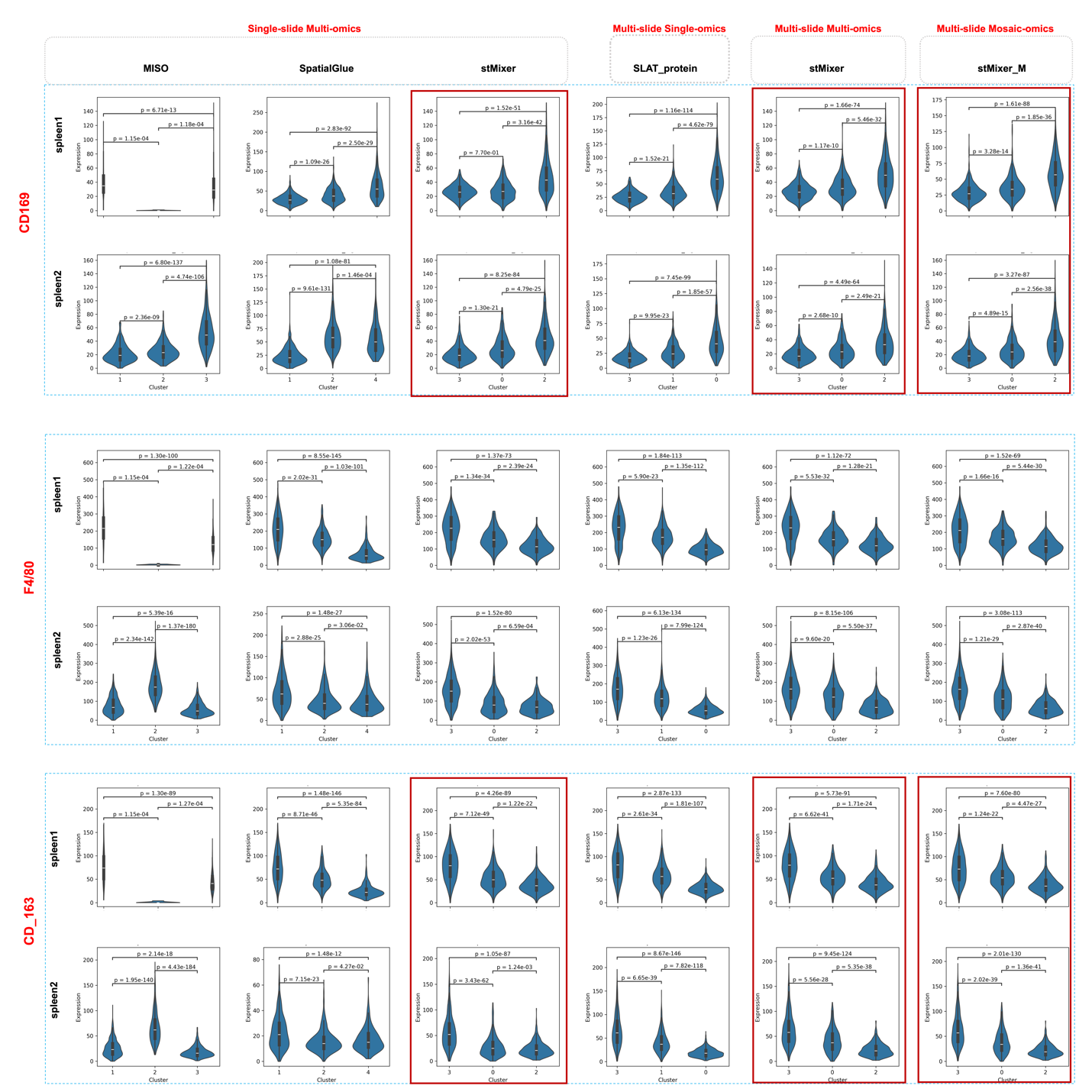


**Supplementary Figure 8.** Box plot showing the protein expression of three marker genes across three macrophage subtypes predicted by six methods. $P-value$ was calculated using the Wilcoxon test between each pair of subtypes.

**
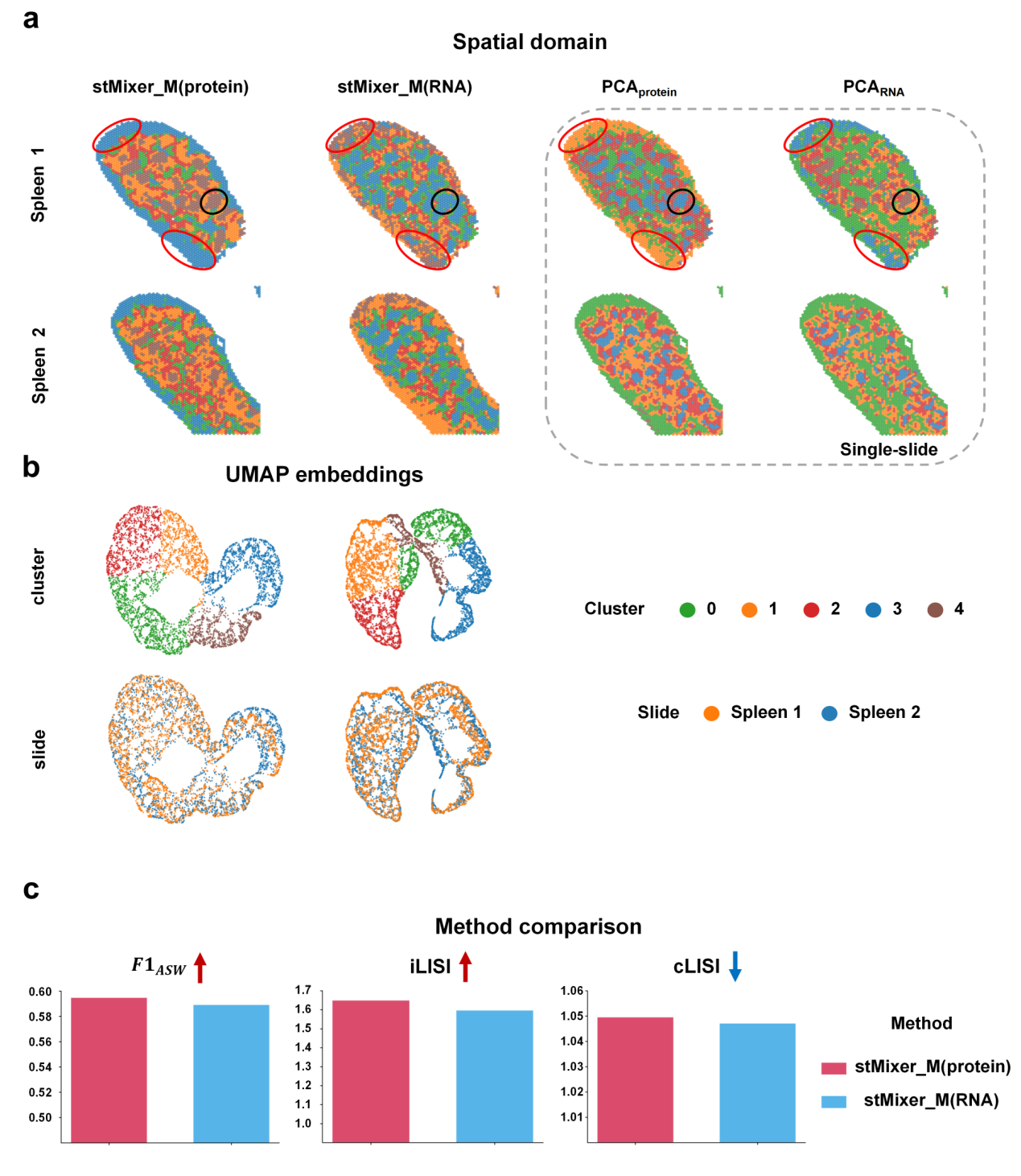
**

**Supplementary Figure 9. Evaluation of RNA and protein as available modalities for cross-slide soft mosaic integration on the mouse spleen dataset**. **a** Spatial domains by stMixer_M(protein), stMixer_M(RNA), and PCA baseline. **b** UMAP embeddings by stMixer_M(protein) and stMixer_M(RNA). **c** Bar plots showing batch-effect correction metrics (${F1}_{ASW}$, iLISI, and cLISI) across stMixer_M(protein) and stMixer_M(RNA).


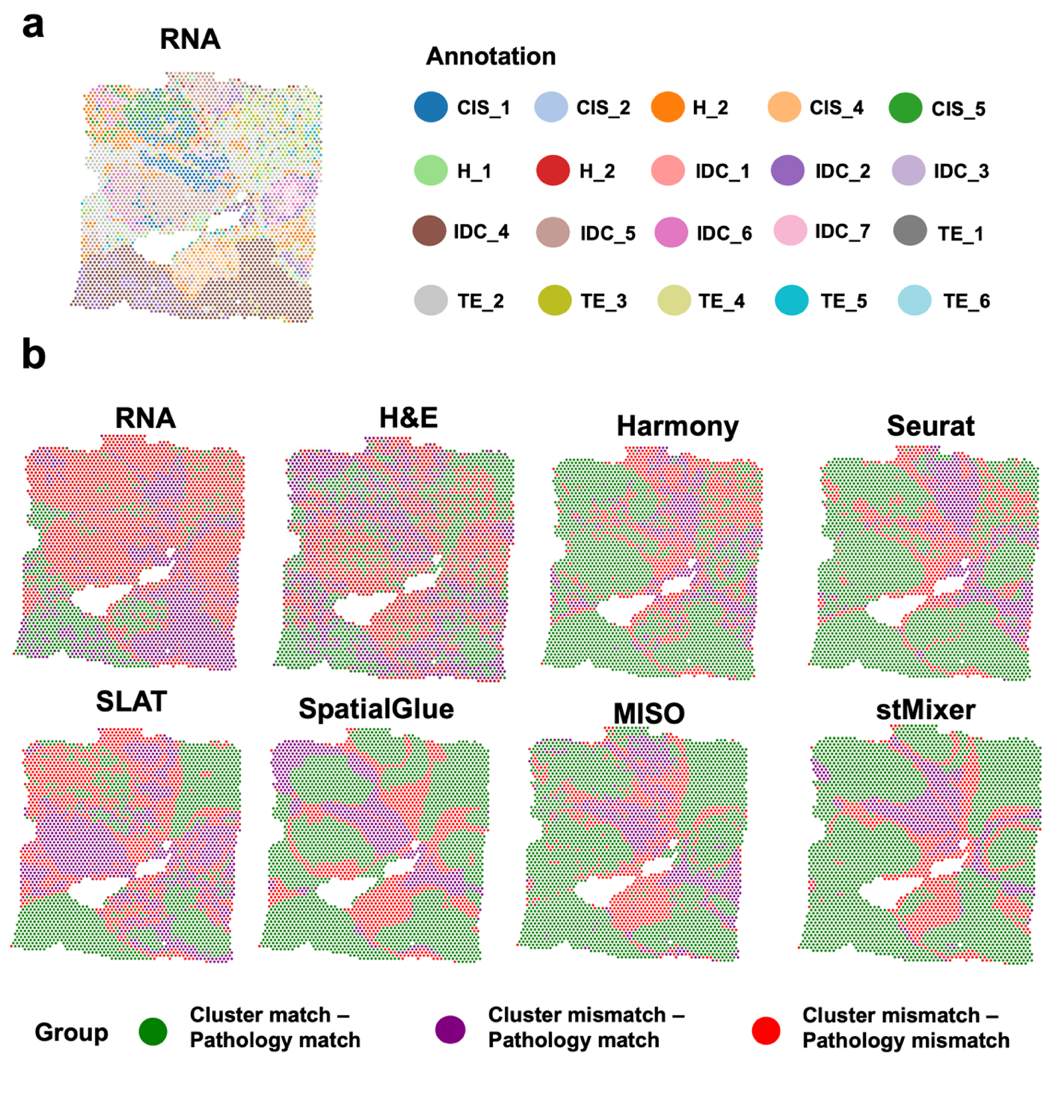


**Supplementary Figure 10**. **Method comparison on the human breast cancer dataset profiled using the Visium spatial transcriptomics platform**. **a** Label transfer from BAS1 to BAS2 slices based on RNA-anchored cell-level voting. Each color indicates one annotated cluster. **b** Spatial classification of each spot based on the agreement between predicted labels from each method and the known histological annotation. Each color indicates one matched group.

**
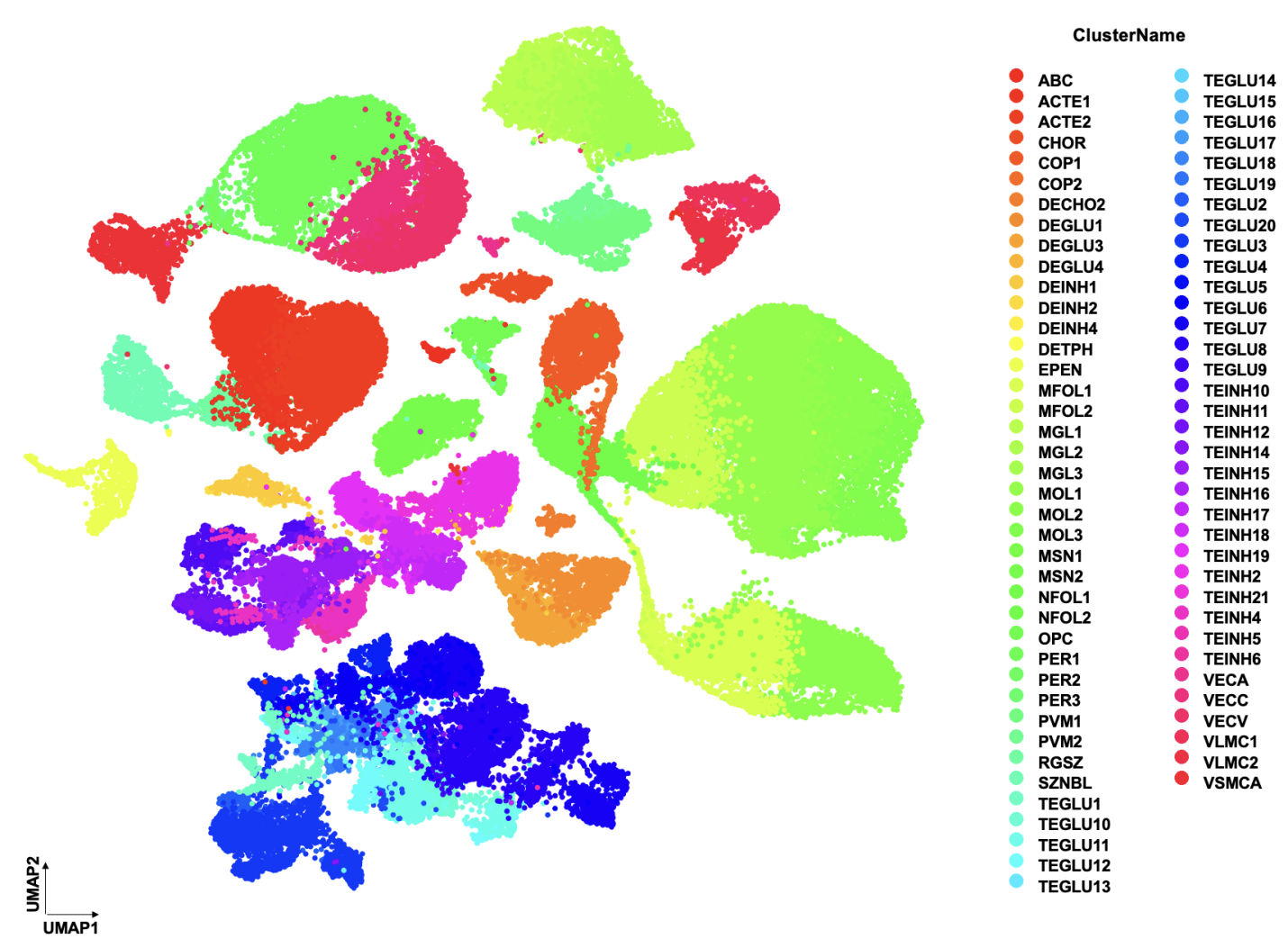
**

**Supplementary Figure 11.** UMAP embedding of 89,689 cells from the mouse brain scRNA-seq reference, colored by 75 different cell types^1^.

**
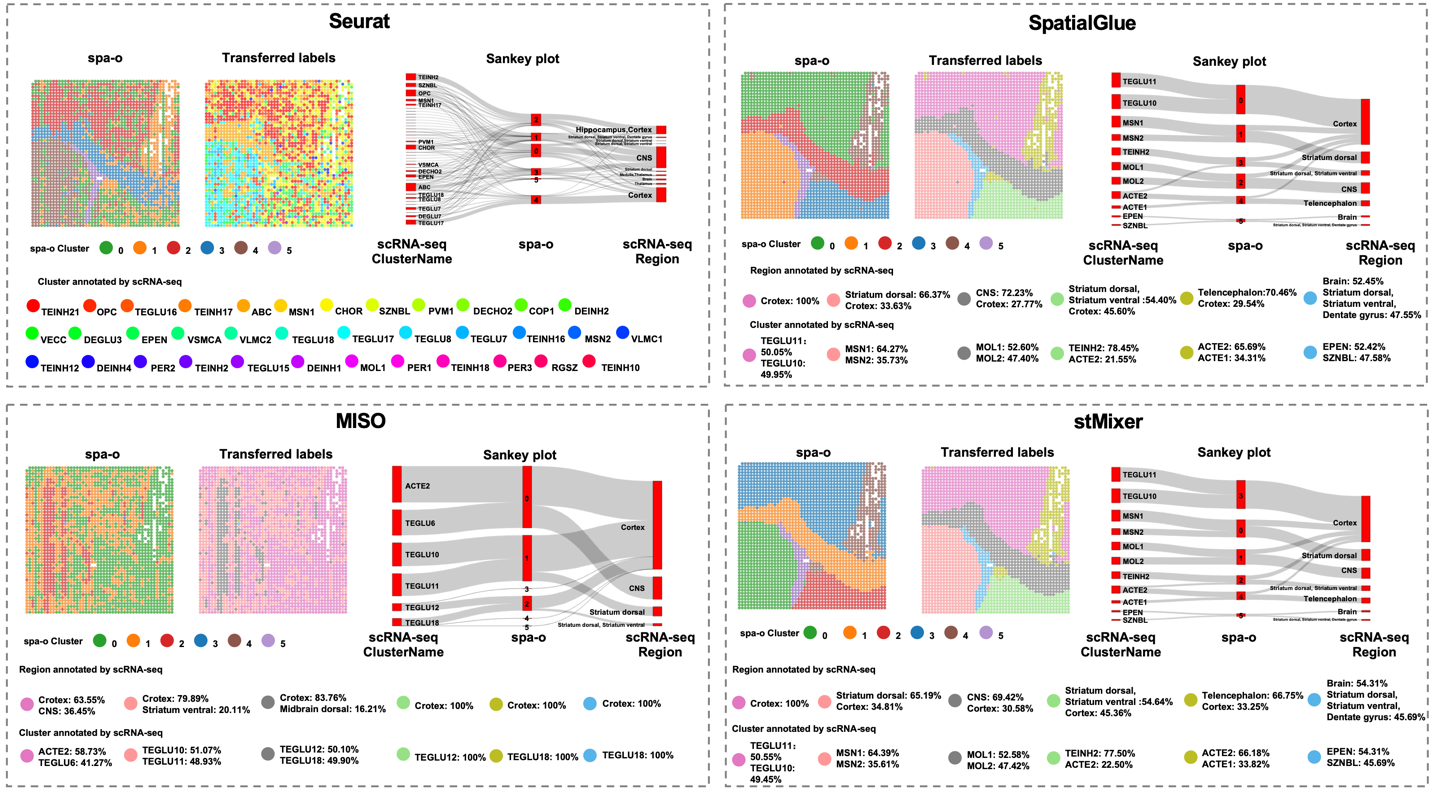
**

**Supplementary Figure 12. Benchmarking of deconvolution performance on spatial multi-omics data using scRNA-seq data reference in a mouse brain 2.** Spatial domains identified by Seurat, MISO, SpatialGlue, and stMixer on spatial multi-omics data (left); corresponding labels transferred from the scRNA-seq data reference (middle); along with a Sankey plot (right) illustrating correspondence between stMixer-inferred spatial domains, scRNA-seq-defined cell types, and anatomical regions (right).

**
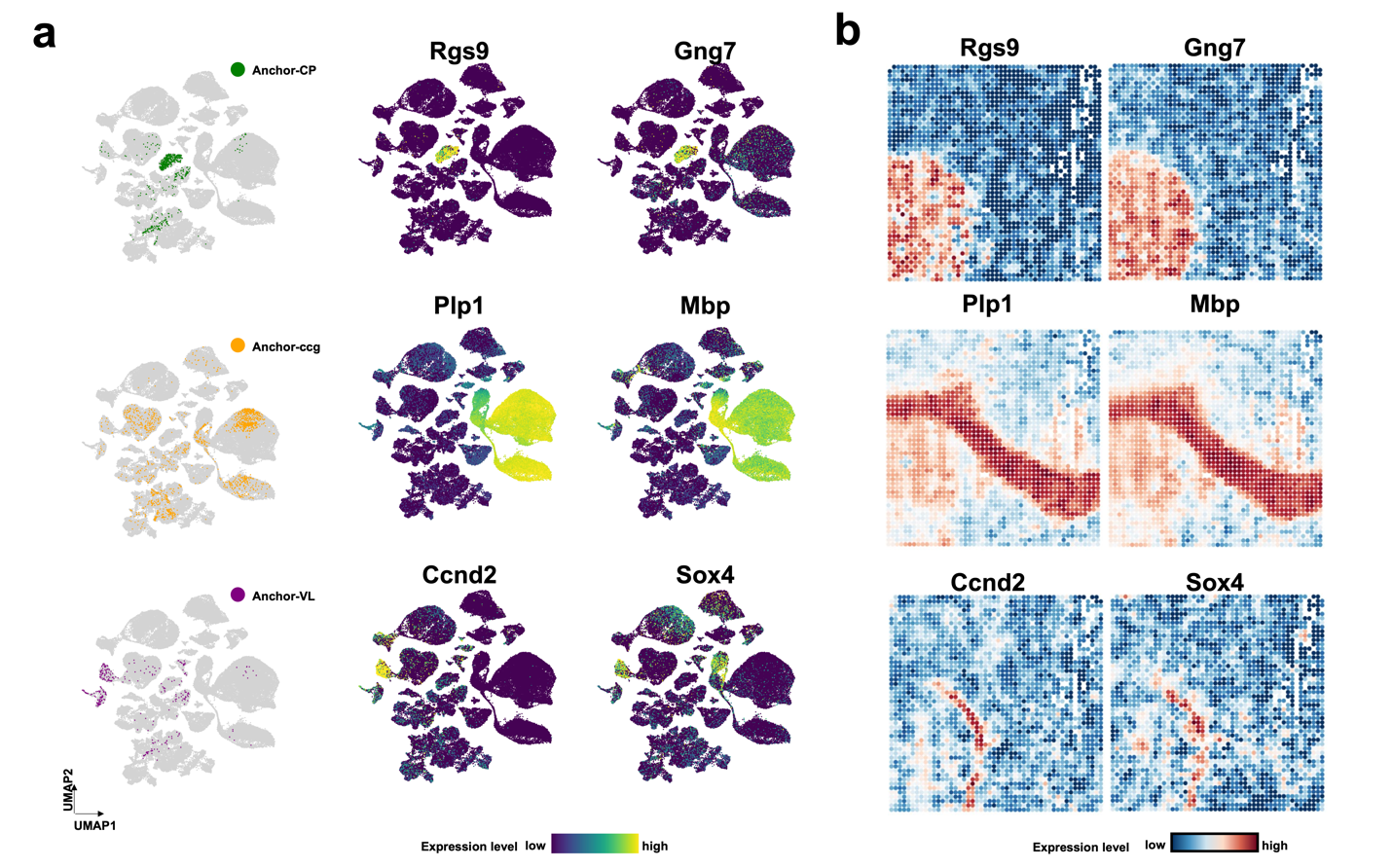
**

**Supplementary Figure 13. a** UMAP visualization of anchor cells in the CP, ccg, and VL regions inferred by MNN matching on the scRNA-seq reference (left) and the expression of six marker genes (*Rgs9*, *Gng7*, *Plp1*, *Mbp, Ccnd2*, and *Sox4*) on the scRNA-seq reference (right). **b** Spatial expression maps of six marker genes (*Rgs9*, *Gng7*, *Plp1*, *Mbp, Ccnd2*, and *Sox4*) in the mouse brain 2.


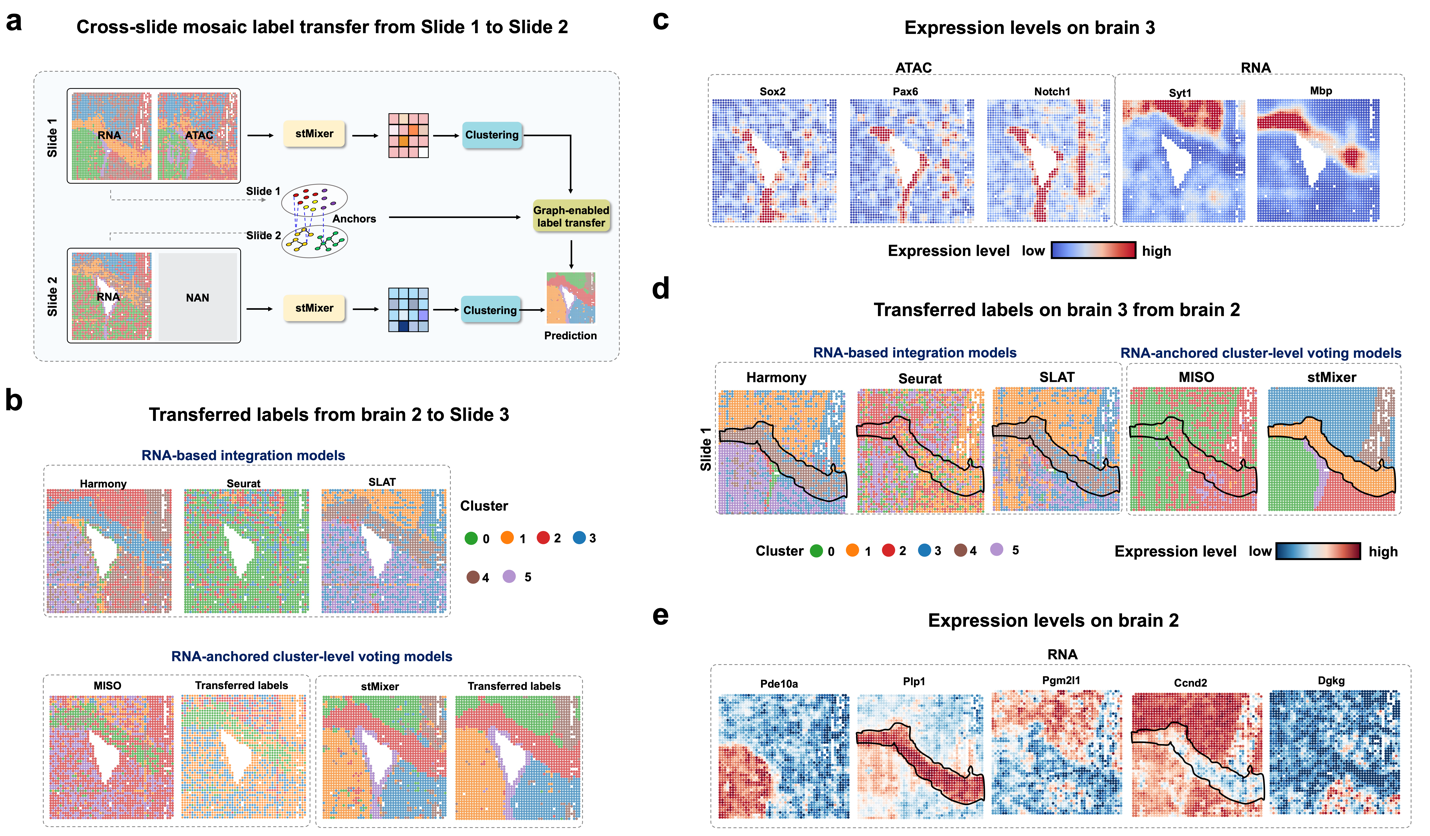


**Supplementary Figure 14. Benchmarking of cross-slide mosaic label transfer on two mouse brain slides. a** Workflow of cross-slide mosaic label transfer from Slide 1 to Slide 2. **b** Spatial domains of transferred labels from brain 2 to brain 3 using five different methods. To facilitate comparison between MISO and stMixer, we also include the clustering results predicted by each method. **c** Spatial expression maps of five marker genes (*Sox2*, *Pax6*, *Notch1*, *Syt1*, and *Mbp*) in the mouse brain 3. **d** Spatial domains of transferred labels from brain 3 to brain 2 using five methods. **e** Spatial expression maps of five marker genes (*Pde10a*, *Plp1*, *Pgm211*, *Ccnd2*, and *Dgkg*) in the mouse brain 2.


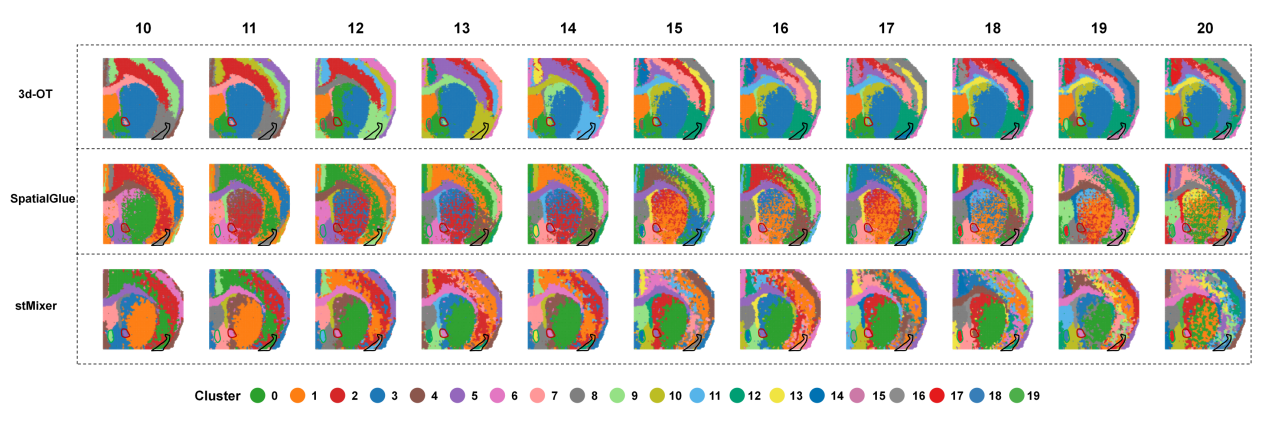


**Supplementary Figure 15. Results under different numbers of clusters on the mouse brain dataset profiled using Spatial ATAC-RNA-seq.** Spatial domains identified by stMixer, SpatialGlue, and 3d-OT on the spatial ATAC–RNA-seq mouse brain dataset across different cluster-number settings ($k=10-20$).


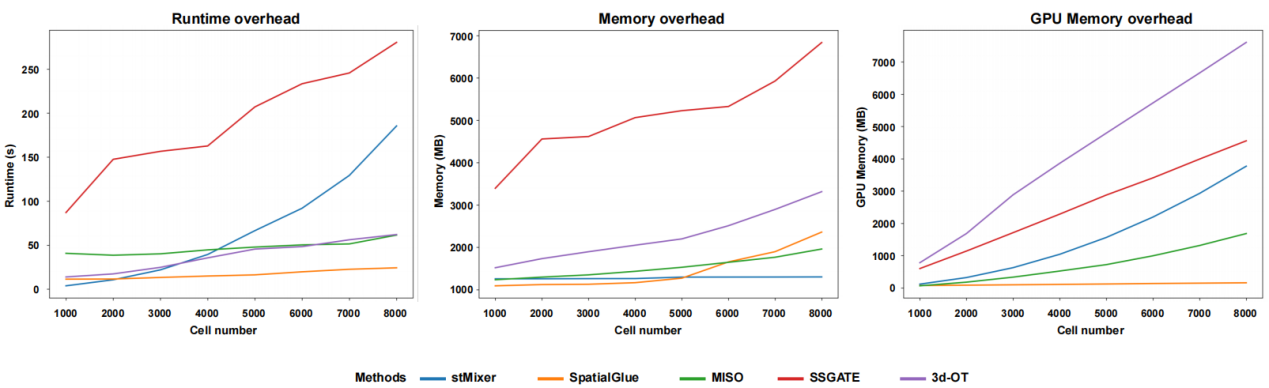


**Supplementary Figure 16.** Comparison of running time and memory usage for the training of five methods including stMixer, 3d-OT, SpatialGlue, MISO, and SSGATE, the different numbers of cells of the simulated data. The experiments were tested on a GPU server with an NVIDIA GeForce RTX 4090 GPU addressing 24 GB.


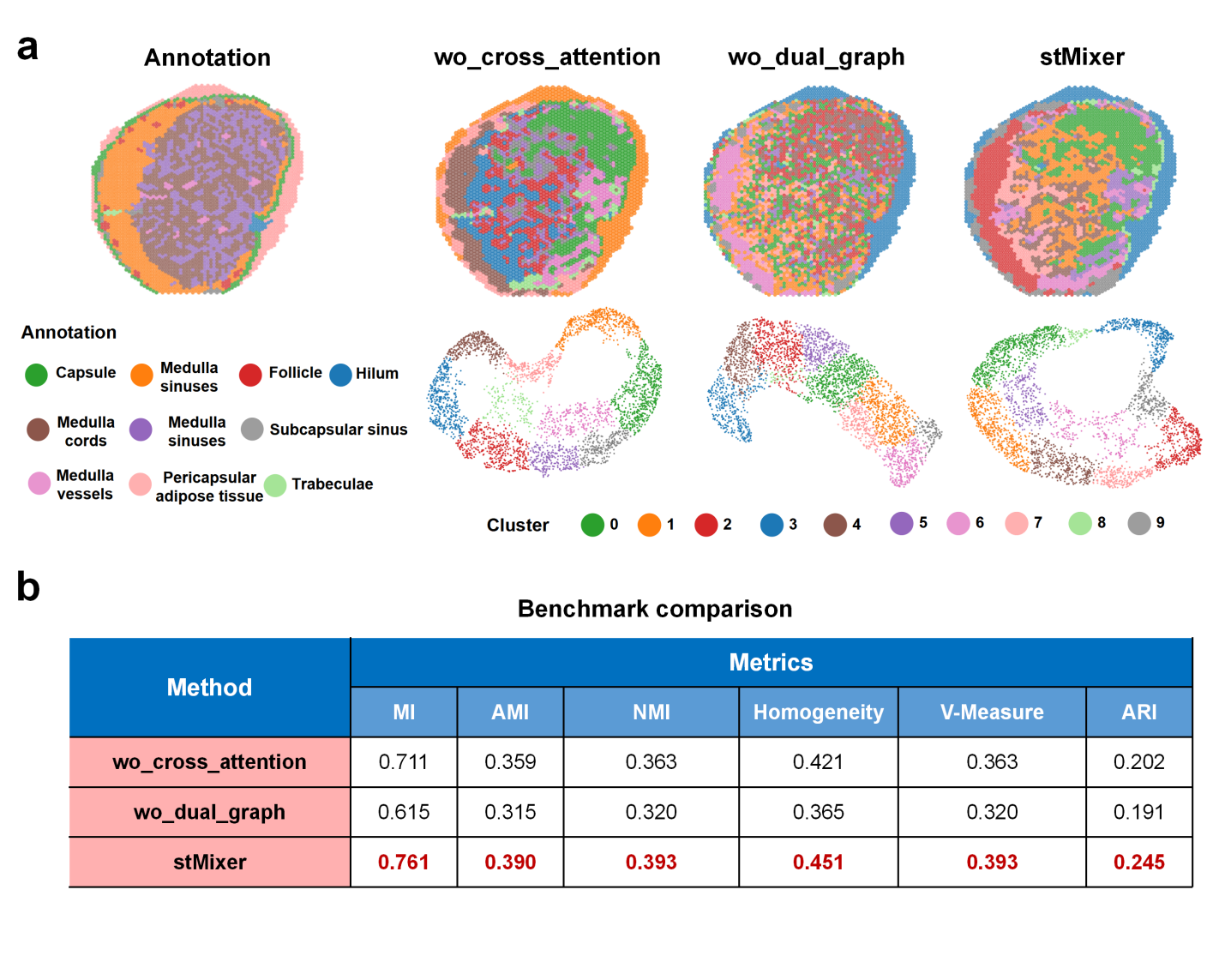


**Supplementary Figure 17. Ablation analysis of the cross-attention fusion and dual-graph self-supervision modules for learning multimodal latent features on the human lymph node dataset. a** Spatial domains and UMAP embeddings by stMixer, stMixer without cross-attention fusion module (wo_cross_attention), and stMixer without dual-graph supervision module(wo_dual-graph). Ground-truth labels are provided for reference. **b** Table showing clustering metrics for stMixer, stMixer without cross-attention fusion module (wo_cross_attention), and stMixer without dual-graph supervision module(wo_dual-graph).


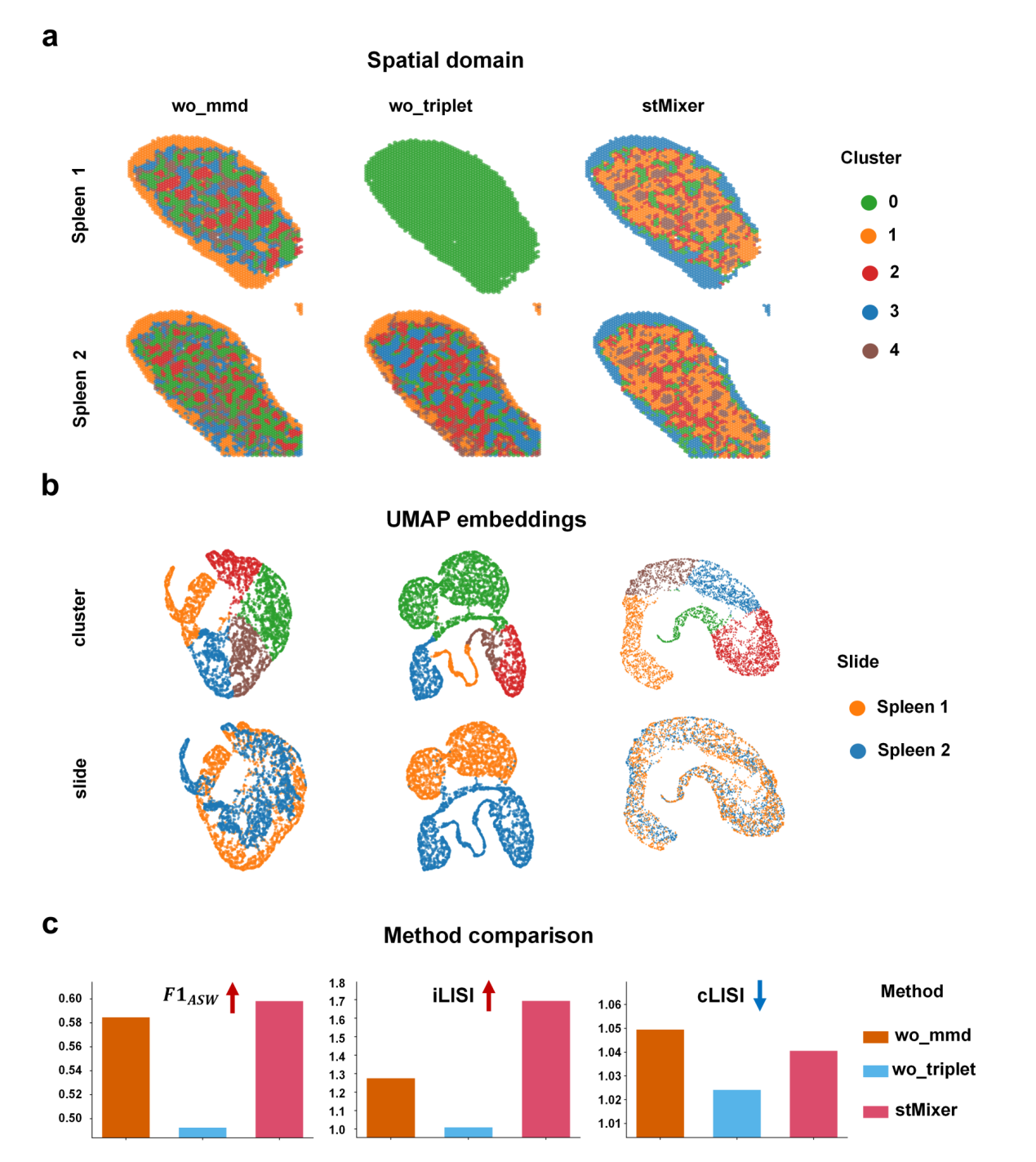


**Supplementary Figure 18. Ablation analysis of the MMD and** **triplet loss modules for cross-slide alignment on the mouse spleen dataset**. **a** Spatial domains by stMixer, stMixer without MMD module (wo_MMD), and stMixer without triplet loss module (wo_triplet). **b** UMAP embeddings by stMixer, stMixer without MMD module (wo_MMD), and stMixer without triplet loss module (wo_triplet). **c** Bar plots comparing batch-effect correction metrics (${F1}_{ASW}$, iLISI, and cLISI) across stMixer, stMixer without MMD module (wo_MMD), and stMixer without triplet loss module (wo_triplet).


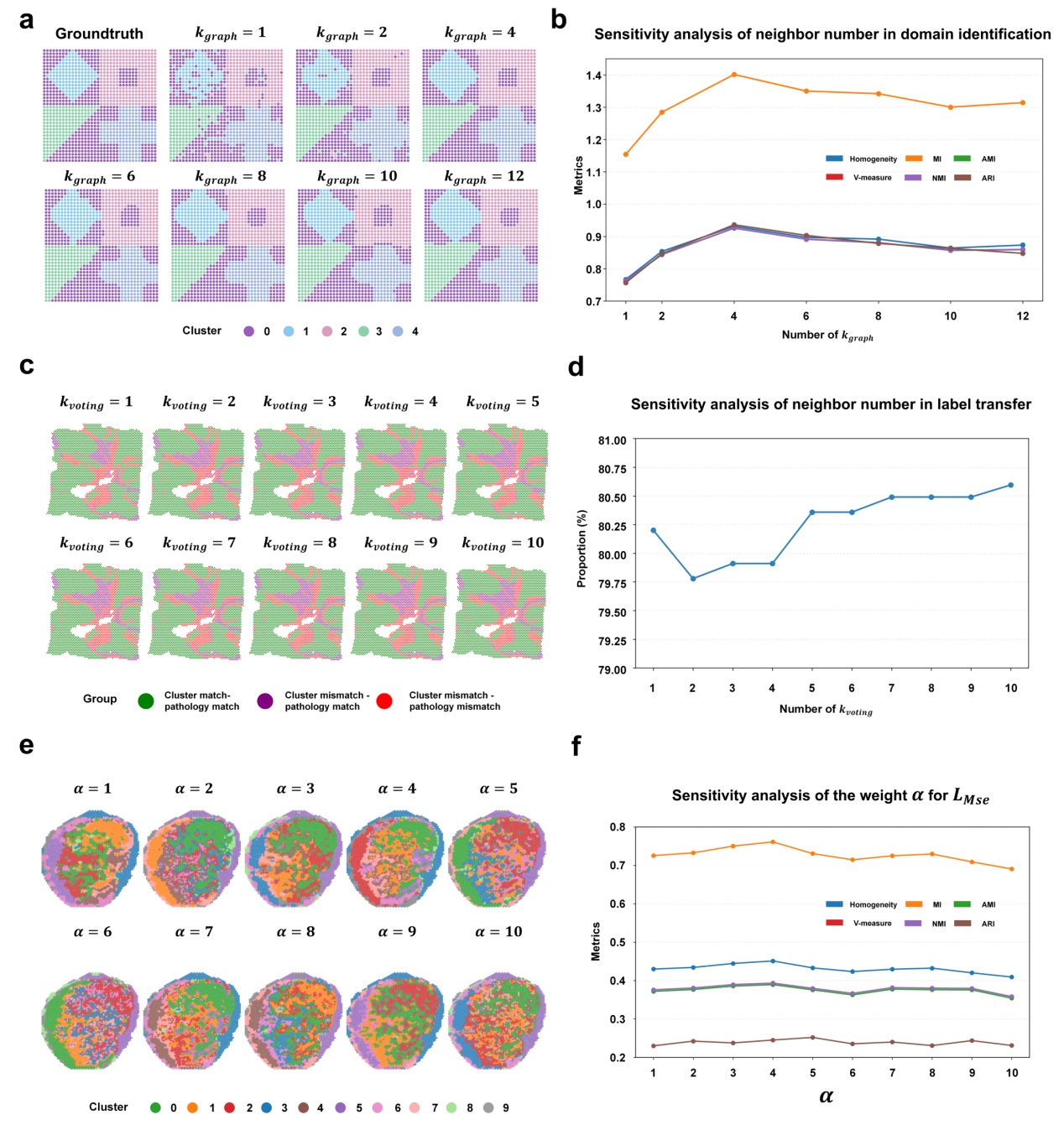


**Supplementary Figure 19. Sensitivity analysis of key hyperparameters. a** Comparison between the identified spatial domains and the ground-truth annotation under different graph-construction neighbor numbers ($k_{graph}=1,2,4,8,10,12$). **b** Sensitivity analysis of the graph-construction neighbor number, showing the trends of six clustering evaluation metrics as $k_{graph}$ varies. **c** Spatial classification of each spot under different $k_{voting}$ settings, based on the agreement between predicted labels and the known histological annotation. Each color indicates one matched group. **d** Changes in the spot-level agreement rate under different $k_{voting}$ settings. **e** Identified spatial domains obtained by varying the weight $\alpha$ for $L_{Mse}$ from 1 to 10. **f** Sensitivity analysis of the weight $\alpha$ for $L_{Mse}$, showing the trends of six clustering evaluation metrics as $\alpha$ varies.


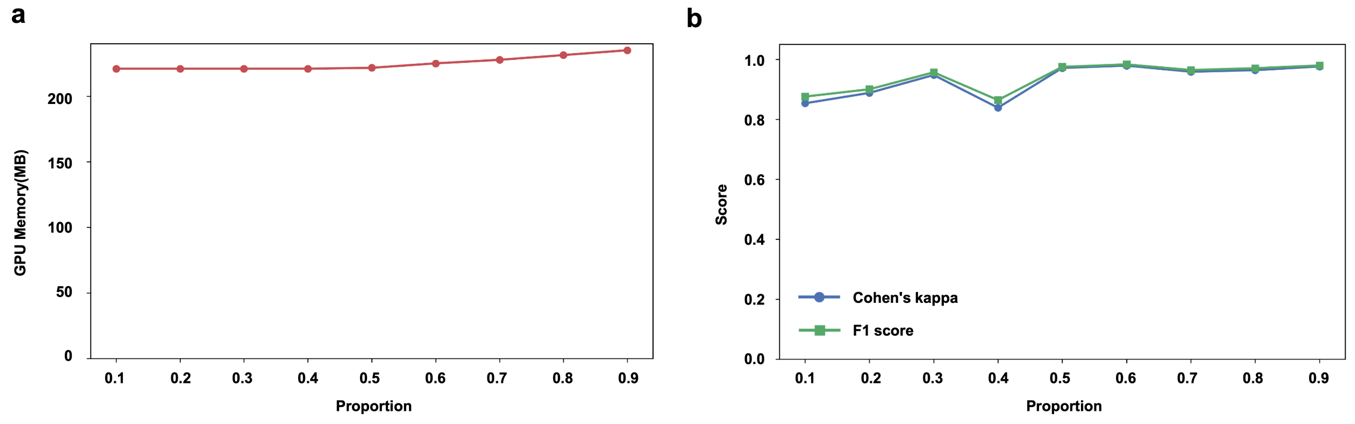


**Supplementary Figure 20.** Benchmark comparison of cell subsampling strategies for optimizing MMD loss, concerning GPU memory consumption (**a**) and clustering consistency relative to using all cells (**b)**.

**Supplementary Tables**

| **Supplementary Table 1. Benchmark implementation strategy and reference pipelines for each method.** | | | | |
| --- | --- | --- | --- | --- |
| **Task** | **Method(s)** | **Reference pipeline / code** | **Output processing** | **evaluation metrics** |
| **Single-slide integration** | PCA-based | Standard PCA implementation | For each method, embeddings were generated and subsequently clustered using the Leiden algorithm. | MI  AMI  NMI  Homogeneity  V-measure  ARI  ASW  CHI |
|  | Seurat | https://github.com/satijalab/seurat |  |  |
|  | MultiVI | https://github.com/scverse/scvi-tools/ |  |  |
|  | totalVI | https://github.com/YosefLab/totalVI_reproducibility |  |  |
|  | SSGATE | https://github.com/Linliu-Bioinf/SSGATE |  |  |
|  | stMixer | https://github.com/YQX-code/stMixer |  |  |
|  | SpatialGlue | https://github.com/JinmiaoChenLab/SpatialGlue | Embeddings were generated and clustered by Leiden (Original) |  |
|  | MISO | https://github.com/kpcoleman/miso | Embeddings were generated and clustered by K-means (Original) |  |
|  | 3d-OT | https://github.com/dbjzs/3d-OT | Embeddings were generated and clustered by mclust (Original) |  |
| **Cross-slide / mosaic integration** | CCA_RNA;CCA_Protein | Standard CCA implementation | For each method, embeddings were generated and subsequently clustered using the Leiden algorithm. | F1-ASW  iLISI  cLISI |
|  | Seurat | https://github.com/satijalab/seurat |  |  |
|  | SLAT_RNA;SLAT_Protein | https://github.com/gao-lab/SLAT |  |  |
|  | MaxFuse | https://github.com/shuxiaoc/maxfuse |  |  |
|  | stMixer | https://github.com/YQX-code/stMixer |  |  |
| **Label transfer** | Seurat | https://github.com/satijalab/seurat | Native anchor-based label transfer was used to obtain transferred labels. | ACC |
|  | Harmony | https://github.com/immunogenomics/harmony | For methods that output query-side representations, clusters, or domain labels, the same voting procedure was used to generate comparable transferred labels. |  |
|  | SLAT | https://github.com/gao-lab/SLAT |  |  |
|  | SpatialGlue | https://github.com/JinmiaoChenLab/SpatialGlue |  |  |
|  | MISO | https://github.com/kpcoleman/miso |  |  |
|  | stMixer | https://github.com/YQX-code/stMixer |  |  |
| Note: For each method, we preserved its task-specific workflow whenever possible. When a method did not directly output the labels required for evaluation, a unified downstream procedure was applied to generate comparable results. All methods within the same task were evaluated using the same task-specific metrics. | | | | |

| **Supplementary Table 2. Dataset-specific target cluster numbers and reference sources used for dynamic Leiden clustering.** | | | |
| --- | --- | --- | --- |
| **Dataset** | **Figure** | **Cluster number** | **Selection criteria** |
| Human lymph node | Fig. 2e | 10 | Ground-truth annotation^2^ |
| Mouse thymus | Fig. 3a | 5 | Original publication^3^ |
| Mouse brain 1 | Fig. 3e | 14 | Original publication^4^ |
| Mouse spleen | Fig. 4b | 5 | Original publication^5^ |
| Human breast cancer | Fig. 5b | 20 | Reference annotation^6^ |
| Mouse brain 2 | Fig. 5d | 6 | Original publication^4^ |
| Mouse brain 3 | Fig. S12b | 6 | Original publication^4^ |
| Note: For each dataset, the target cluster number was determined according to the original publication, ground-truth annotation, or reference annotation. The same target cluster number was used for all methods compared on the same dataset. The Leiden resolution was dynamically adjusted to reach the predefined target cluster number. | | | |

**References**

1 Zeisel, A. *et al.* Molecular Architecture of the Mouse Nervous System. *Cell* **174**, 999–1014.e22 (2018). <https://doi.org/10.1016/j.cell.2018.06.021>.

2 Long, Y. *et al.* Deciphering spatial domains from spatial multi-omics with SpatialGlue. *Nature Methods* **21**, 1658–1667 (2024). <https://doi.org/10.1038/s41592-024-02316-4>.

3 Liao, S. *et al.* Integrated Spatial Transcriptomic and Proteomic Analysis of Fresh Frozen Tissue Based on Stereo-seq. *bioRxiv*, 2023.04.28.538364 (2023). <https://doi.org/10.1101/2023.04.28.538364>.

4 Zhang, D. *et al.* Spatial epigenome–transcriptome co-profiling of mammalian tissues. *Nature* **616**, 113–122 (2023). <https://doi.org/10.1038/s41586-023-05795-1>.

5 Ben-Chetrit, N. *et al.* Integration of whole transcriptome spatial profiling with protein markers. *Nature Biotechnology* **41**, 788–793 (2023). <https://doi.org/10.1038/s41587-022-01536-3>.

6 Xu, H. *et al.* Unsupervised spatially embedded deep representation of spatial transcriptomics. *Genome Medicine* **16**, 12 (2024). <https://doi.org/10.1186/s13073-024-01283-x>.
